# Identification of lysophosphatidic acid in serum as a factor that promotes epithelial apical junctional complex organization

**DOI:** 10.1016/j.jbc.2022.102426

**Published:** 2022-08-27

**Authors:** Shotaro Sakakibara, Ayuko Sakane, Takuya Sasaki, Masakazu Shinohara, Tomohiko Maruo, Muneaki Miyata, Kiyohito Mizutani, Yoshimi Takai

**Affiliations:** 1 Department of Biochemistry, Tokushima University Graduate School of Medicine, Tokushima, Japan; 2 Division of Pathogenetic Signaling, Department of Biochemistry and Molecular Biology, Kobe University Graduate School of Medicine, Kobe, Japan; 3 Department of Interdisciplinary Researches for Medicine and Photonics, Institute of Post-LED Photonics, Tokushima University, Tokushima, Japan; 4 Division of Epidemiology, Kobe University Graduate School of Medicine, Kobe, Japan; 5 The Integrated Center for Mass Spectrometry, Kobe University Graduate School of Medicine, Kobe, Japan

**Keywords:** apical junctional complex, adherens junctions, tight junctions, cadherin, catenin, afadin, LPA, Ab, antibody, AJ, adherens junction, AJC, apical junctional complex, BIM1, bisindolylmaleimide 1, CAM, cell adhesion molecule, CIAP, calf intestinal alkaline phosphatase, CNF, cytotoxic necrotizing factor, cPKC, conventional PKC, DAG, diacylglycerol, DMEM, Dulbecco's modified Eagle's medium, F-actin, filamentous actin, FABP, F-actin–binding protein, FBS, fetal bovine serum, JAM, junctional adhesion molecule, LPA, lysophosphatidic acid, LPAR, LPA receptor, MDCK, Madin–Darby canine kidney, nPKC, novel PKC, PA, phosphatidic acid, PLC, phospholipase C, S1P, sphingosine-1-phosphate, TJ, tight junction, TPA, 12-*O*-tetradecanoylphorbol 13-acetate, VE, vascular endothelial

## Abstract

The apical junctional complex (AJC) consists of adherens junctions (AJs) and tight junctions and regulates epithelial integrity and remodeling. However, it is unclear how AJC organization is regulated based on environmental cues. We found here using cultured EpH4 mouse mammary epithelial cells that fetal bovine serum (FBS) in a culture medium showed an activity to promote AJC organization and that FBS showed an activity to promote tight junction formation even in the absence of AJ proteins, such as E-cadherin, αE-catenin, and afadin. Furthermore, we purified the individual factor responsible for these functions from FBS and identified this molecule as lysophosphatidic acid (LPA). In validation experiments, purified LPA elicited the same activity as FBS. In addition, we found that the AJC organization–promoting activity of LPA was mediated through the LPA receptor 1/5 *via* diacylglycerol–novel PKC and Rho–ROCK pathway activation in a mutually independent, but complementary, manner. We demonstrated that the Rho–ROCK pathway activation–mediated AJC organization was independent of myosin II-induced actomyosin contraction, although this signaling pathway was previously shown to induce myosin II activation. These findings are in contrast to the literature, as previous results suggested an AJC organization–disrupting activity of LPA. The present results indicate that LPA in serum has an AJC organization–promoting activity in a manner dependent on or independent of AJ proteins.

In epithelial cells, the apical junctional complex (AJC) consists of adherens junctions (AJs) and tight junctions (TJs) and attaches neighboring cells to each other to form a sheet (1). The TJs localize at the apical side of the AJs along the lateral plasma membrane, establishing epithelial apicobasal polarity (1, 2, 3). The AJs play a role in the establishment of mechanical connections between neighboring cells to maintain the epithelial integrity and regulate morphological changes of the epithelial sheet (4). The TJs function as an epithelial barrier that prevents the diffusion of soluble molecules across the epithelial sheet and as a boundary between the apical and basolateral membrane domains to produce their polarization (2, 5, 6). The AJs regulate TJ formation and maintenance, thus organizing and maintaining the AJC (7). In this article, a term “AJC organization” is used to refer to the AJ and TJ structure formation.

Major epithelial AJ cell adhesion molecules (CAMs) are cadherin, especially E-cadherin, and nectin (8, 9), whereas major epithelial TJ CAMs are claudin and junctional adhesion molecule (JAM) (2, 6, 10). Occludin and tricellulin are also the components of TJs, although they are not typical CAMs (11, 12). The AJC, particularly the AJs, is heavily undercoated with two types of filamentous actin (F-actin) structure, the mesh-like F-actin structure just beneath the plasma membrane and the circumferential F-actin structure, which locates slightly far from the plasma membrane (13, 14, 15). Both F-actin structures bind myosin II to form actomyosin bundles, regulating cell adhesion strength and plasticity of the AJs (14, 16).

Both F-actin structures are associated with many F-actin–binding proteins (FABPs), which localize at two distinct regions, just beneath the plasma membrane and slightly far from the plasma membrane (17). The former group of FABPs anchors the mesh-like F-actin structure to the plasma membrane, whereas the latter group of FABPs bind to both the mesh-like and circumferential F-actin structures to connect them. One group of FABPs just beneath the plasma membrane binds to AJ and TJ CAMs. This group includes αE-catenin, afadin, and ZO-1/2/3: αE-catenin binds to E-cadherin through β-catenin and afadin binds to nectin at the AJs, whereas ZO-1/2/3 bind to claudin, occludin, JAM, and tricellulin at the TJs (2, 8, 9).

We previously showed using mainly Madin–Darby canine kidney (MDCK) epithelial cells that the *trans*-interaction of nectin first initiates cell adhesion and recruits E-cadherin to the nectin-mediated cell adhesion sites by the binding of afadin to αE-catenin, enhancing the *trans*-interaction of E-cadherin and AJ formation (18, 19, 20, 21, 22). We recently found using EpH4 mouse mammary epithelial cells that afadin regulates the undercoating of circumferential F-actin structure by binding to αE-catenin complexed with β-catenin and enhancing its FAB activity and its association with the AJs, eventually strengthening E-cadherin-mediated cell adhesion (23). We also showed using mainly MDCK cells that during and/or after AJ formation, *trans*-interacting nectin enhances the recruitment of first JAM and then claudin and occludin to the apical side of the AJs by the binding of afadin to ZO-1 and enhances TJ formation, eventually establishing the AJC (24, 25).

A Ca^2+^ switch assay was frequently used to investigate the mechanisms for AJC organization because cadherin is a Ca^2+^-dependent CAM (7, 26). This assay was performed by preculturing epithelial cells at a low Ca^2+^ concentration, which is typically a micromolar level, followed by reculturing them at a physiological millimolar Ca^2+^ concentration. Most previous studies on the AJC organization, including Ca^2+^ switch experiments, were performed using epithelial cells cultured in the serum-containing medium, and the roles of each of the AJ and TJ components in the AJC organization were investigated (20, 25, 27, 28, 29, 30, 31, 32, 33). However, it could not be determined from these previous experiments using epithelial cells cultured in the serum-containing medium whether AJC organization was dependent on Ca^2+^ alone, serum alone, or both Ca^2+^ and serum.

We investigated here the role and modes of action of fetal bovine serum (FBS) in AJC organization using EpH4 cells cultured in the FBS-containing or FBS-free medium and found that FBS in a culture medium showed an activity to promote AJC organization and that FBS showed an activity to promote TJ formation even in the absence of AJ proteins, such as E-cadherin, αE-catenin, and afadin. Furthermore, we purified the individual factor responsible for these functions from FBS and identified this molecule as lysophosphatidic acid (LPA). LPA is involved in various cellular functions, including wound healing, proliferation, migration, differentiation, and survival, through its receptors (34). The concentration of LPA in serum is frequently increased in inflammation, development, and progression of various cancers (35). These previous results suggested an AJC organization–disrupting activity of LPA, and the present findings that LPA has an AJC organization–promoting activity are in contrast to these previous results. We showed here that LPA in FBS has an AJC organization–promoting activity in a manner dependent on or independent of AJ proteins through the LPA receptor (LPAR) 1/5 *via* diacylglycerol (DAG)–novel PKC (nPKC) and Rho–ROCK pathway activation. FBS is hereafter simply referred to as serum.

## Results

### AJC organization–promoting activity of serum in WT EpH4 cells in a Ca^2+^ switch assay

**Figure 1:**
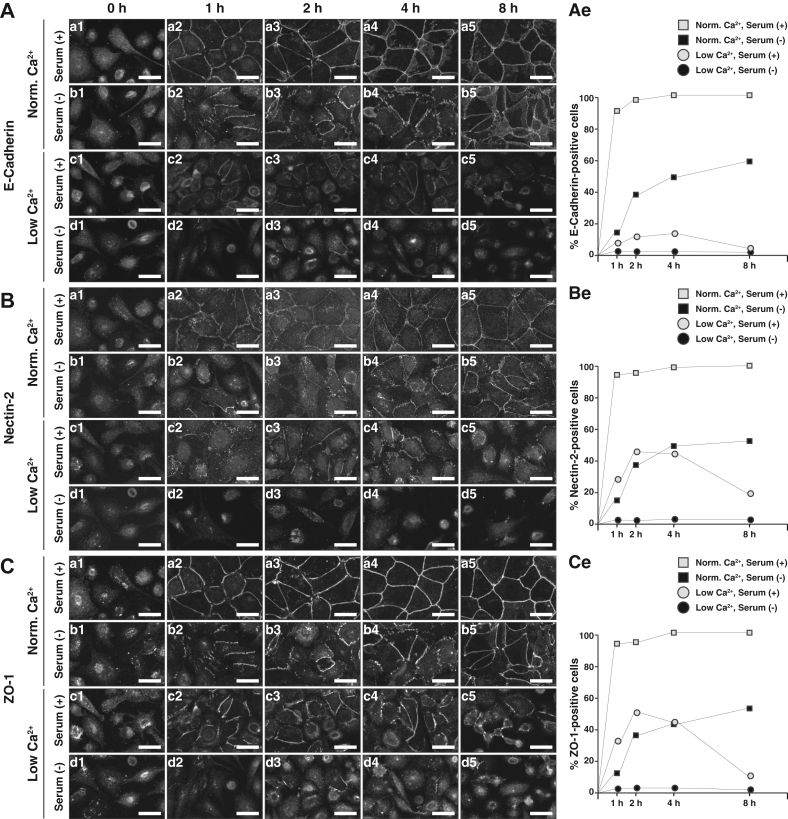
**AJC organization–promoting activity of serum in WT EpH4 cells.** WT EpH4 cells precultured at 5 mM EGTA in the serum-free medium for 3 h were recultured either in the presence or the absence of 5 mM EGTA in the serum-containing medium or the serum-free medium for 0, 1, 2, 4, or 8 h. The cells were fixed and stained with an anti-E-cadherin Ab (*A*), an anti-nectin-2 Ab (*B*), and an anti-ZO-1 Ab (*C*), and then observed by immunofluorescence microscopy. Quantitative analyses are shown in the *right panels*. The percentages of the cells positive for the E-cadherin, nectin-2, or ZO-1 signal at the cell–cell boundaries at various time points are shown. Serum (+) indicates the serum-containing medium, whereas serum (−) indicates the serum-free medium; Normal Ca^2+^ indicates reculturing the cells in the absence of EGTA, whereas low Ca^2+^ indicates reculturing the cells in the presence of 5 mM EGTA, respectively; and scale bars indicate 25 μm. The results are representative of three independent experiments. Ab, antibody; AJC, apical junctional complex.

We first examined whether serum affects AJC organization in WT EpH4 cells in the Ca^2+^ switch assay. In the following Ca^2+^ switch assay, the medium with a low Ca^2+^ concentration was prepared by adding 5 mM EGTA Ca^2+^ chelator to the medium containing 1.8 mM CaCl_2_, whereas the medium with a physiological Ca^2+^ concentration was prepared by using the medium containing 1.8 mM CaCl_2_. This Ca^2+^-chelated condition and this physiological Ca^2+^ concentration are hereafter referred to simply “low Ca^2+^” and “normal Ca^2+^,” respectively. In WT EpH4 cells precultured at low Ca^2+^ in the serum-free medium, the E-cadherin, nectin-2, or ZO-1 signal was hardly accumulated at the cell–cell boundaries (Fig. 1, *A*–*C*). When these cells were recultured at normal Ca^2+^ in the serum-containing medium, these signals were accumulated at the cell–cell boundaries in a time-dependent manner, consistent with the previous observations (27) (Fig. 1, Aa1–Aa5, Ae, Ba1–Ba5, Be, Ca1–Ca5, and Ce). In contrast, when the cells were recultured at normal Ca^2+^ in the serum-free medium, the E-cadherin, nectin-2, and ZO-1 signals were accumulated at the cell–cell boundaries in a time-dependent manner, but their kinetics were much slower than those in the cells recultured at normal Ca^2+^ in the serum-containing medium (Fig. 1, Ab1–Ab5, Ae, Bb1–Bb5, Be, Cb1–Cb5, and Ce). These signals were fully accumulated after a longer period of incubation (Fig. S1*A*). These results indicate that serum shows a strong activity to promote the Ca^2+^ readdition–induced accumulation of E-cadherin, nectin-2, and ZO-1 at the cell–cell boundaries although it does not alter the steady state of their accumulation on the plateau.

When WT EpH4 cells were recultured at low Ca^2+^ in the serum-free medium, the E-cadherin, nectin-2, or ZO-1 signal was hardly accumulated at the cell–cell boundaries at any periods (Fig. 1, Ad1–Ad5, Ae, Bd1–Bd5, Be, Cd1–Cd5, and Ce). In contrast, when the cells were recultured at low Ca^2+^ in the serum-containing medium, the nectin-2 and ZO-1 signals were accumulated at the cell–cell boundaries in a time-dependent manner (Fig. 1, Ac1–Ac5, Ae, Bc1–Bc5, Be, Cc1–Cc5, and Ce). The E-cadherin signal was slightly accumulated at the cell–cell boundaries in a time-dependent manner under this condition, although E-cadherin does not *trans*-interact with each other to induce cell adhesion at low Ca^2+^ (7, 26). The exact mechanism for this slight accumulation of the E-cadherin signal is not known, but the probable mechanism is that E-cadherin did not *trans*-interact with each other to induce cell adhesion at low Ca^2+^, but non-*trans*-interacting E-cadherin might be associated with nectin-2-based cell adhesion because it was previously shown that nectin-1-based cell adhesion is Ca^2+^ independent and non-*trans*-interacting E-cadherin is associated with nectin-1-based cell adhesion in MDCK cells when they were cultured at low Ca^2+^ in the presence of PKC-activating phorbol ester 12-*O*-tetradecanoylphorbol 13-acetate (TPA) (29). These results indicate that serum alone shows an activity to promote the accumulation of nectin-2 and ZO-1 in a manner independent of E-cadherin-based cell adhesion at low Ca^2+^. It was noted that the accumulation of the nectin-2 and ZO-1 signals at the cell–cell boundaries decreased at 8 h after recultured at low Ca^2+^ in the serum-containing medium, indicating that the serum activity to promote the accumulation of nectin-2 and ZO-1 in this experimental system is transient. It was also noted that the number of cells accumulating the nectin-2 and ZO-1 signals under this condition was far less than that under the condition where recultured at normal Ca^2+^ in the serum-containing medium and nearly as much as that under the condition where recultured at normal Ca^2+^ in the serum-free medium (Fig. 1, *B* and *C*). These results indicate that Ca^2+^ alone or serum alone has an activity to promote the accumulation of nectin-2 and ZO-1 to some extent, and the combination of Ca^2+^ and serum has a stronger activity in a cooperative manner.

Other TJ and AJ components were examined in WT EpH4 cells recultured at normal Ca^2+^ or low Ca^2+^ in the serum-containing or serum-free medium, following the preculture at low Ca^2+^ in the serum-free medium. Essentially the same results as that for the E-cadherin signal were obtained for the β-catenin and αE-catenin signals; that for the nectin-2 signal was obtained for the afadin signal; and that for the ZO-1 signal was obtained for the claudin-3 signal (Fig. S1*B*). Collectively, these results indicate that serum shows an activity to promote AJC organization at normal Ca^2+^, whereas serum shows an activity to promote TJ formation, but not AJ formation, even at low Ca^2+^, where E-cadherin does not *trans*-interact with each other to induce cell adhesion. These results revealed a novel activity of serum to promote AJC organization, and that serum has a TJ formation–promoting activity even in the absence of E-cadherin-based cell adhesion.

### TJ formation–promoting activity of serum in *E-cadherin*-KO EpH4 cells

**Figure 2:**
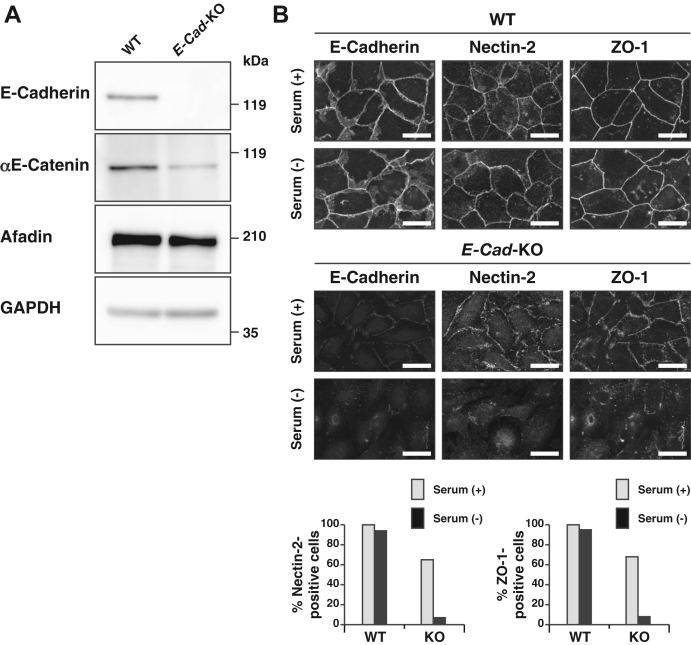
**TJ formation–promoting activity of serum in *E-cadherin*-KO EpH4 cells.***A*, disappearance of the E-cadherin protein in *E-cadherin*-KO EpH4 cells. The cell lysates from the WT and *E-cadherin*-KO EpH4 cells were subjected to SDS-PAGE, followed by Western blotting with the indicated Abs. *B*, activity of serum for the localization of nectin-2 and ZO-1 in *E-cadherin*-KO EpH4 cells. WT or *E-cadherin*-KO EpH4 cells were cultured in the serum-containing or serum-free medium for 2 h. The cells were fixed and stained with the indicated Abs and then observed by immunofluorescence microscopy. Quantitative analyses are shown in the *lower panels*. The percentages of the cells positive for the nectin-2 signal at the cell–cell boundaries in (*B*) are 100% (WT/serum [+], n = 102), 94% (WT/serum [−], n = 101), 65% (*E-cadherin*-KO/serum [+], n = 78), and 7% (*E-cadherin*-KO/serum [−], n = 80). The percentages of the cells positive for the ZO-1 signal at the cell–cell boundaries in (*B*) are 100% (WT/serum [+], n = 184), 95% (WT/serum [−], n = 185), 68% (*E-cadherin*-KO/serum [+], n = 148), and 8% (*E-cadherin*-KO/serum [−], n = 149). Serum (+) indicates the serum-containing medium, whereas serum (−) indicates the serum-free medium; E-Cad indicates E-cadherin; and scale bars indicate 25 μm. The results are representative of three independent experiments. Ab, antibody; TJ, tight junction.

To confirm the TJ formation–promoting activity of serum in the absence of E-cadherin-based cell adhesion, we next examined whether serum showed the same activity in *E-cadherin*-KO EpH4 cells cultured at normal Ca^2+^. The following experiments were conducted at normal Ca^2+^. We generated *E-cadherin*-KO EpH4 cells using CRISPR/Cas9-mediated gene editing. In Western blotting, the protein band of E-cadherin was not observed in this mutant cell (Fig. 2*A*). A single band around 210 kDa was detected for afadin in WT cells as described previously (27), and it was also detected in *E-cadherin*-KO EpH4 cells to a similar extent (Fig. 2*A*). It was noted that the intensity of the protein band of αE-catenin was slightly reduced in *E-cadherin*-KO EpH4 cells, compared with that in WT cells (Fig. 2*A*).

In *E-cadherin*-KO EpH4 cells cultured at normal Ca^2+^ in the serum-containing or serum-free medium, the E-cadherin signal was not observed at the cell–cell boundaries (Fig. 2*B*). Under these conditions, the nectin-2 signal was accumulated at the cell–cell boundaries although its intensity in *E-cadherin*-KO EpH4 cells cultured in the serum-containing medium was more than that in *E-cadherin*-KO EpH4 cells cultured in the serum-free medium (Fig. 2*B*). In *E-cadherin*-KO EpH4 cells cultured at normal Ca^2+^ in the serum-containing medium, the ZO-1 signal was accumulated at the cell–cell boundaries (Fig. 2*B*). However, in *E-cadherin*-KO EpH4 cells cultured at normal Ca^2+^ in the serum-free medium, the ZO-1 signal was not accumulated at the cell–cell boundaries but localized randomly inside the cells with irregular shapes (Fig. 2*B*). Essentially the same results as that for the E-cadherin signal were obtained for the β-catenin and αE-catenin signals; that for the nectin-2 signal was obtained for the afadin signal; and that for the ZO-1 signal was obtained for the claudin-3 signal (Fig. S2*A*). WT EpH4 cells cultured in the serum-containing or serum-free medium did not show any changes in the E-cadherin, nectin-2, or ZO-1 signal (Fig. 2*B*). Collectively, these results indicate that serum has a TJ formation–promoting activity in the absence of E-cadherin-based cell adhesion.

### TJ formation–promoting activity of serum in *αE-catenin*-KO EpH4 cells

**Figure 3:**
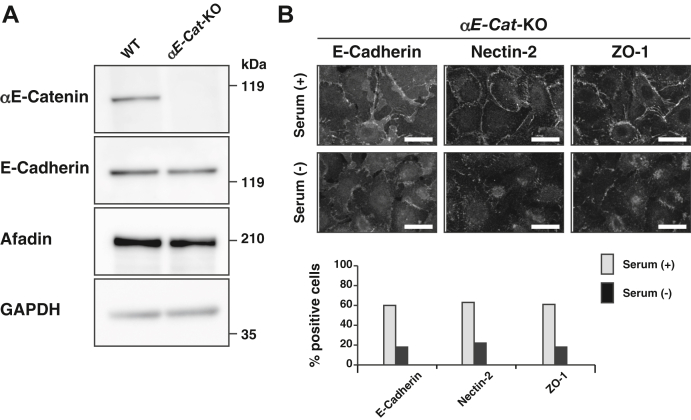
**TJ formation–promoting activity of serum in *αE-catenin*-KO EpH4 cells.***A*, disappearance of the αE-catenin protein in *αE-catenin*-KO EpH4 cells. The cell lysates from the WT and *αE-catenin*-KO EpH4 cells were subjected to SDS-PAGE, followed by Western blotting with the indicated Abs. *B*, activity of serum for the localization of E-cadherin, nectin-2, and ZO-1 in *αE-catenin*-KO EpH4 cells. *αE-catenin*-KO EpH4 cells were cultured in the serum-containing or serum-free medium for 2 h. The cells were fixed and stained with the indicated Abs and then observed by immunofluorescence microscopy. Quantitative analyses are shown in the *lower panel*. The percentages of the cells positive for the E-cadherin signal at the cell–cell boundaries in (*B*) are 60% (serum [+], n = 53) and 18% (serum [−], n = 80). The percentages of the cells positive for the nectin-2 signal at the cell–cell boundaries in (*B*) are 63% (serum [+], n = 76) and 22% (serum [−], n = 61). The percentages of the cells positive for the ZO-1 signal at the cell–cell boundaries in (*B*) are 61% (serum [+], n = 129) and 18% (serum [−], n = 141). Serum (+) indicates the serum-containing medium, whereas serum (−) indicates the serum-free medium; αE-cat indicates αE-catenin; and scale bars indicate 25 μm. The results are representative of three independent experiments. Ab, antibody; TJ, tight junction.

αE-Catenin is essential for E-cadherin to organize the AJC (9, 33, 36). We further confirmed the TJ formation–promoting activity of serum in a manner independent of E-cadherin-based cell adhesion using *αE-catenin*-KO EpH4 cells cultured at normal Ca^2+^. We generated *αE-catenin*-KO EpH4 cells using CRISPR/Cas9-mediated gene editing. In Western blotting, the protein band of αE-catenin was not observed in this mutant cell (Fig. 3*A*). The protein band of afadin was detected in both WT and *αE-catenin*-KO EpH4 cells to a similar extent. Essentially the same results as those obtained for *E-cadherin*-KO EpH4 cells were obtained for *αE-catenin*-KO EpH4 cells (Figs. 3*B* and S3*A*) and indicate that serum has a TJ formation–promoting activity in the absence of E-cadherin-based cell adhesion.

### AJC organization–promoting activity of serum in *afadin*-KO EpH4 cells

**Figure 4:**
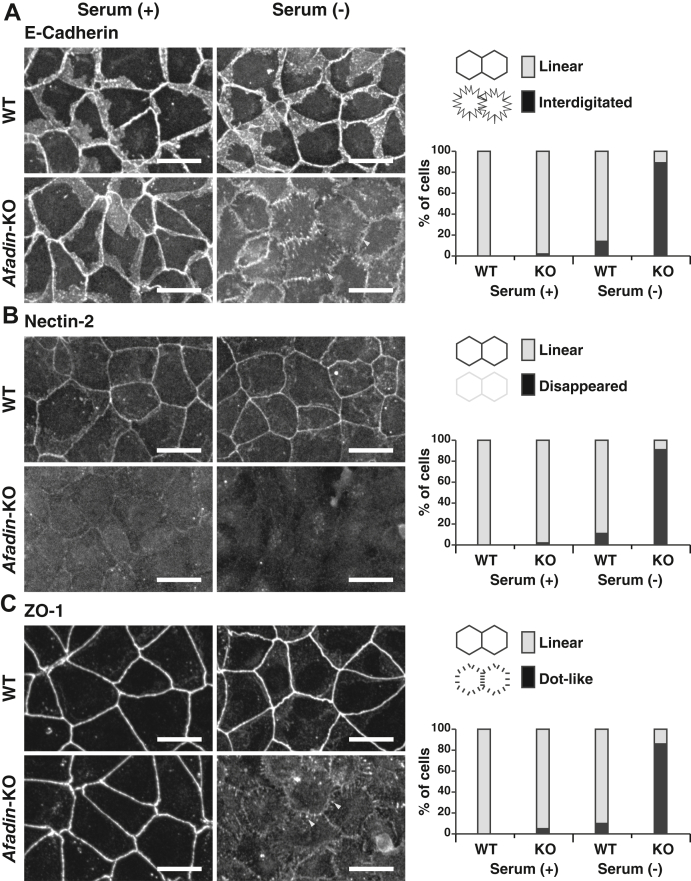
**AJC organization–promoting activity of serum in a manner that is independent of and mostly complementary to afadin.** WT or *afadin*-KO EpH4 cells were cultured in the serum-containing or serum-free medium for 8 h. The cells were fixed and stained with the indicated Abs and then observed by immunofluorescence microscopy. *Yellow arrowheads* shown in (*A*) and (*C*) indicate the interdigitated E-cadherin signal pattern and the dot-like ZO-1 signal pattern, respectively. Quantitative analyses are shown in the *right panels*. The percentages of the cells with the interdigitated E-cadherin signal pattern in (*A*) are 0% (WT/serum [+], n = 185), 2% (*afadin*-KO/serum [+], n = 192), 14% (WT/serum [−], n = 187), and 89% (*afadin*-KO/serum [−], n = 182). The percentages of the cells with the disappeared nectin-2 signal pattern in (*B*) are 0% (WT/serum [+], n = 195), 2% (*afadin*-KO/serum [+], n = 185), 11% (WT/serum [−], n = 189), and 91% (*afadin*-KO/serum [−], n = 175). The percentages of the cells with the dot-like ZO-1 signal pattern in (*C*) are 0% (WT/serum [+], n = 190), 5% (*afadin*-KO/serum [+], n = 172), 10% (WT/serum [−], n = 194), and 86% (*afadin*-KO/serum [−], n = 191). Serum (+) indicates the serum-containing medium, whereas serum (−) indicates the serum-free medium; and scale bars indicate 25 μm. The results are representative of three independent experiments. Ab, antibody; AJC, apical junctional complex.

Afadin is involved in AJC organization cooperatively with αE-catenin (8). Because serum has a TJ formation–promoting activity even in the absence of AJ proteins, such as E-cadherin and αE-catenin, we further examined whether serum shows the similar TJ formation–promoting activity in *afadin*-KO EpH4 cells. The following experiments were conducted at normal Ca^2+^. We used *afadin*-KO EpH4 cells generated previously (27). In *afadin*-KO EpH4 cells cultured in the serum-containing medium, the E-cadherin signal was accumulated at the cell–cell boundaries, and its shape was similar to that observed in WT EpH4 cells cultured in the serum-containing or serum-free medium (Fig. 4*A*). However, in *afadin*-KO EpH4 cells cultured in the serum-free medium, the E-cadherin signal was slightly reduced in its intensity and changed from a linear pattern to an interdigitated pattern (Fig. 4*A*). In *afadin*-KO EpH4 cells cultured in the serum-containing medium, the nectin-2 signal at the cell–cell boundaries was reduced in its intensity compared with that in WT EpH4 cells cultured in the serum-containing medium. Moreover, the nectin-2 signal completely disappeared in *afadin-*KO EpH4 cells cultured in the serum-free medium, whereas it was not changed by the serum depletion in WT EpH4 cells (Fig. 4*B*). In *afadin*-KO EpH4 cells cultured in the serum-containing medium, the ZO-1 signal was accumulated at the cell–cell boundaries as a linear pattern and the shape of this ZO-1 signal was similar to that observed in WT EpH4 cells cultured in the serum-containing or serum-free medium (Fig. 4*C*). However, in *afadin*-KO EpH4 cells cultured in the serum-free medium, the ZO-1 signal was not accumulated at the cell–cell boundaries as a linear pattern but remained as a dot-like pattern (Fig. 4*C*). Essentially the same results as that for the E-cadherin signal were obtained for the β-catenin and αE-catenin signals; and that for the ZO-1 signal was obtained for the claudin-3 (Fig. S4, *A*–*D*). Collectively, these results indicate that serum has an AJC organization–promoting activity in the absence of afadin, confirming the AJC organization–promoting activity of serum. Because afadin enhances the kinetics of the cell adhesion based on the E-cadherin–β-catenin–αE-catenin complex, leading to AJ formation (27), these results indicate that serum has a TJ formation–promoting activity in a manner independent of afadin-enhanced E-cadherin–β-catenin–αE-catenin complex–based cell adhesion in the Ca^2+^ switch assay. It was also noted that *afadin*-KO EpH4 cells cultured in the serum-free medium showed reduced cell surface area compared with WT EpH4 cells cultured in the serum-containing or serum-free medium or *afadin*-KO EpH4 cells cultured in the serum-containing medium, indicating that afadin and serum have an activity for making the cells flatten out in a complementary manner.

We previously showed that afadin acts as an upstream regulator of AJC organization (18, 20, 25, 37). Afadin enhances the recruitment of the αE-catenin–β-catenin–E-cadherin complex to AJs (18, 20, 23). Afadin also enhances the kinetics of TJ formation (23, 25). In the context of the afadin-promoted TJ formation, AJ formation is a prerequisite (23, 25), suggesting that monitoring ZO-1 can be an effective way to predict the presence of other AJC components at the cell–cell boundaries. Therefore, in the following experiments, we used *afadin*-KO EpH4 cells to investigate the detailed roles and mechanisms of serum in the activity to promote AJC organization by monitoring the changes of the ZO-1 signal.

### Time-dependent and reversible AJC organization–promoting activity of serum in *afadin*-KO EpH4 cells

**Figure 5:**
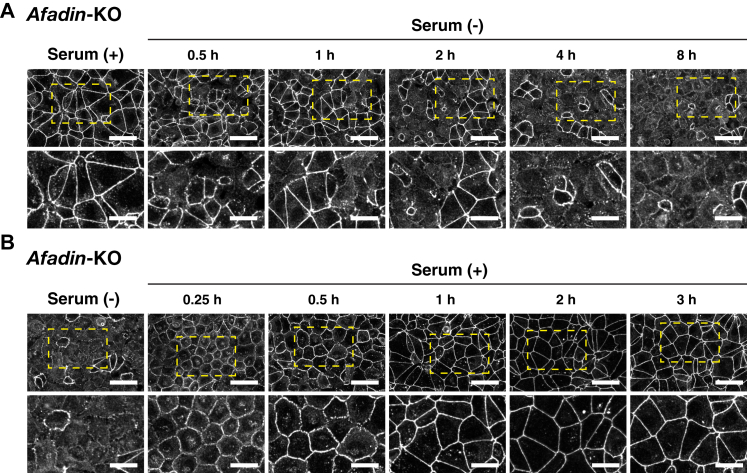
**Time-dependent AJC disruption and restoration by serum depletion and readdition, respectively, in *afadin*-KO EpH4 cells.***A*, time-dependent AJC disruption in *afadin*-KO EpH4 cells cultured in the serum-free medium. *Afadin*-KO EpH4 cells were cultured in the serum-containing or serum-free medium for 0.5, 1, 2, 4, or 8 h. The cells were fixed and stained with the anti-ZO-1 Ab and then observed by immunofluorescence microscopy. The *boxed regions* in *upper panels* are highlighted in *lower panels*. The scale bars indicate 50 μm (*upper panels*) and 25 μm (*lower panels*). *B*, time-dependent AJC restoration by serum readdition in *afadin*-KO EpH4 cells precultured in the serum-free medium. *Afadin*-KO EpH4 cells were precultured in the serum-free medium for 8 h and then cultured in the serum-containing medium for 0, 0.25, 0.5, 1, 2, or 3 h. The cells were fixed and stained with the anti-ZO-1 Ab and then observed by immunofluorescence microscopy. The *boxed regions* in *upper panels* are highlighted in *lower panels*. Serum (+) indicates the serum-containing medium, whereas serum (−) indicates the serum-free medium; and scale bars indicate 50 μm (*upper panels*) and 25 μm (*lower panels*). The results are representative of three independent experiments. Ab, antibody; AJC, apical junctional complex.

We then examined whether the serum-promoted AJC organization is time-dependent and reversible. The changes of the ZO-1 signal observed in *afadin*-KO EpH4 cells cultured at normal Ca^2+^ in the serum-free medium were time-dependent and reversible (Fig. 5, *A* and *B*). The ZO-1 signal at the cell–cell boundaries in *afadin*-KO EpH4 cells started to decrease at 0.5 h after the serum depletion, followed by a gradual decrease, and finally disappeared at 8 h (Fig. 5*A*). Such phenotypes were observed in only 10% of the cells at 0.5 h after the serum depletion but in 90% of the cells at 8 h after the serum depletion. In the remaining 10% of the cells, the ZO-1 signal was still observed as a single and linear pattern at the cell–cell boundaries (Fig. S5). On the other hand, the ZO-1 signal changed by the 8 h serum depletion was gradually restored after the serum addition to the serum-free medium (Fig. 5*B*). The serum addition also restored the reduced cell surface area change shown in *afadin*-KO EpH4 cells cultured in the serum-free medium. These results indicate that the serum-promoted AJC organization is time-dependent and reversible.

### No requirement of afadin or serum for the formation of the αE-catenin–β-catenin–E-cadherin complex at AJs

In an attempt to uncover the mechanism for the AJC disruption in *afadin*-KO cells in response to the serum depletion, we addressed the possibility that the dissociation of the αE-catenin–β-catenin–E-cadherin complex underlies the AJC disruption. We previously showed that afadin is not required for the formation of the αE-catenin–β-catenin–E-cadherin complex at AJs in EpH4 cells cultured at normal Ca^2+^ in the serum-containing medium (23). We examined whether E-cadherin, β-catenin, and αE-catenin form a complex in *afadin*-KO EpH4 cells cultured at normal Ca^2+^ in the serum-free medium. When αE-catenin was immunoprecipitated using the anti-αE-catenin antibody (Ab) from the lysates of WT and *afadin*-KO EpH4 cells cultured at normal Ca^2+^ in the serum-free medium, β-catenin and E-cadherin were coimmunoprecipitated with αE-catenin to similar extents (Fig. S6). These results indicate that neither medium serum nor afadin is required for the formation of the αE-catenin–β-catenin–E-cadherin complex at AJs, ruling out the possibility that the dissociation of the αE-catenin–β-catenin–E-cadherin complex underlies the AJC disruption.

### Identification of LPA as an AJC organization–promoting factor in serum

**Figure 6:**
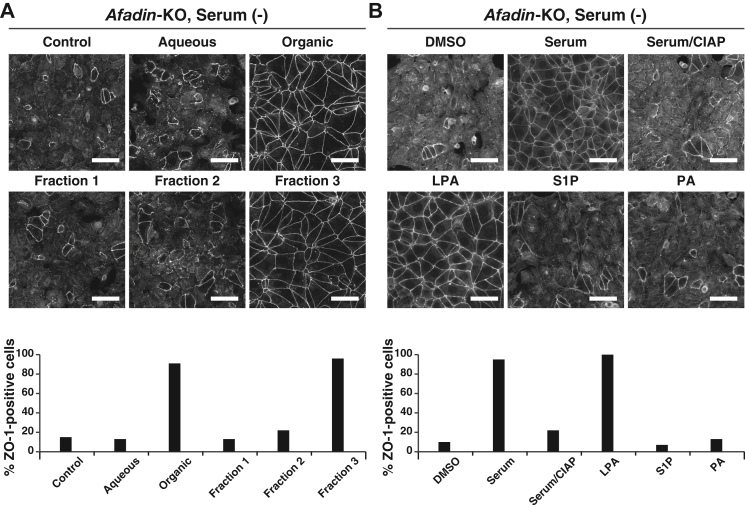
**Involvement of LPA in the serum-promoted AJC organization.***A*, purification of the active factor for AJC organization from serum. *Afadin*-KO EpH4 cells were cultured in the serum-free medium in the presence of 1% BSA with or without the aqueous phase fraction, the organic phase fraction, fraction 1 (the neutral lipid fraction), fraction 2 (the fatty acid fraction), or fraction 3 (the phospholipid/lysophospholipid/glycolipid fraction) for 8 h. The cells were fixed and stained with the anti-ZO-1 Ab and then observed by immunofluorescence microscopy. Quantitative analysis is shown in the *lower panel*. The percentages of the ZO-1-positive cells are 15% (control, n = 176), 13% (aqueous phase, n = 210), 91% (organic phase, n = 184), 13% (fraction 1, n = 177), 22% (fraction 2, n = 186), and 96% (fraction 3, n = 211). *B*, identification of LPA as the active factor in serum for AJC organization. *Afadin*-KO EpH4 cells were cultured in the serum-free medium in the presence of 1% BSA with DMSO, 2% serum, 2% CIAP-treated serum, 10 μM LPA, 10 μM S1P, or 10 μM PA for 8 h. The cells were fixed and stained with the anti-ZO-1 Ab and then observed by immunofluorescence microscopy. Quantitative analysis is shown in the *lower panel*. The percentages of the ZO-1-positive cells are 10% (DMSO, n = 208), 95% (serum, n = 212), 22% (serum/CIAP, n = 191), 100% (LPA, n = 191), 7% (S1P, n = 204), and 13% (PA, n = 180). Serum (−) indicates the serum-free medium; aqueous and organic indicate the aqueous phase and the organic phase, respectively; and scale bars indicate 50 μm. The results are representative of three independent experiments. Ab, antibody; AJC, apical junctional complex; BSA, bovine serum albumin; CIAP, calf intestinal alkaline phosphatase; DMSO, dimethyl sulfoxide; LPA, lysophosphatidic acid; PA, phosphatidic acid; S1P, sphingosine-1-phosphate.

We next attempted to identify the individual factor(s) responsible for these functions from serum. After several trials, we subjected serum to the Bligh and Dyer method (38). The activity was detected in the organic phase, but not in the aqueous phase (Fig. 6*A*), suggesting that a lipid mediator(s) accounts for the serum activity. The organic phase was subjected to a solid-phase C18 cartridge to separate lipids into three fractions: fraction 1, neutral lipid fraction; fraction 2, fatty acid fraction; and fraction 3, phospholipid/lysophospholipid/glycolipid fraction. The activity was mostly recovered in the phospholipid/lysophospholipid/glycolipid fraction but not the neutral lipid fraction or the fatty acid fraction (Fig. 6*A*). In addition, treatment of serum with calf intestinal alkaline phosphatase (CIAP), which hydrolyzes phosphatidic acid (PA), LPA, or sphingosine-1-phosphate (S1P) to remove the phosphoryl group (39), resulted in the loss of the AJC organization–promoting activity of serum in *afadin*-KO EpH4 cells cultured at normal Ca^2+^ in the serum-free medium (Fig. 6*B*).

We therefore examined whether LPA, S1P, and PA obtained from commercial sources substituted for serum to promote AJC organization in *afadin*-KO EpH4 cells cultured at normal Ca^2+^ in the serum-free medium. LPA, but not S1P or PA, restored the ZO-1 signal at the cell–cell boundaries in *afadin*-KO EpH4 cells cultured in the serum-free medium, and this activity was similar to that of serum (Figs. 6*B* and 4). LPA restored the ZO-1 signal at the cell–cell boundaries not only in *afadin*-KO EpH4 cells cultured in the serum-free medium but also in WT EpH4 cells in the Ca^2+^ switch assay and in *E-cadherin*-KO and *αE-catenin*-KO EpH4 cells cultured at normal Ca^2+^ in the serum-free medium (Figs. S1*C*, S2*B*, S3*B*, S7 and Figure 1, Figure 2, Figure 3, Figure 4). It was also noted that LPA restored the reduced cell surface area change shown in *afadin*-KO EpH4 cells cultured in the serum-free medium. These activities of LPA were similar to those of serum (Figure 1, Figure 2, Figure 3, Figure 4). Collectively, these results indicate that LPA is the active factor of serum that promotes AJC organization.

### AJC organization–promoting activity of serum and LPA through LPAR 1 and 5

**Figure 7:**
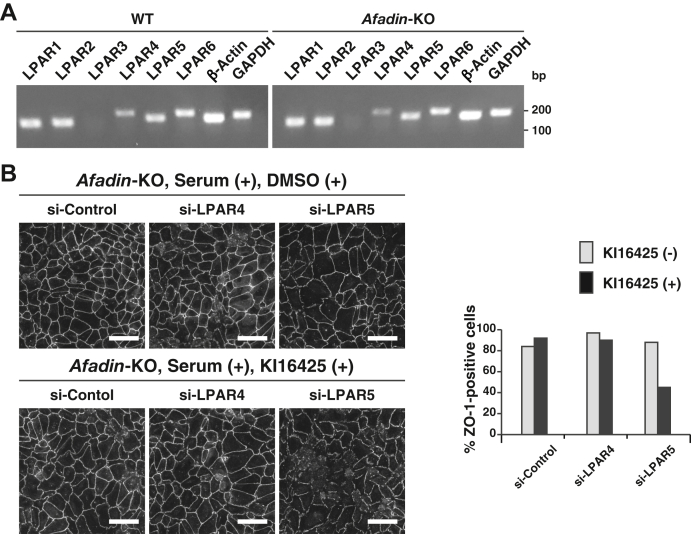
**Involvement of LPAR in the serum-promoted AJC organization.***A*, the gene-expression profiles of LPARs in WT and *afadin*-KO EpH4 cells. Total RNAs extracted from WT and *afadin*-KO EpH4 cells were subjected to RT–PCR, followed by ethidium bromide–stained agarose gel electrophoresis. *B*, identification of LPAR1/5 as the receptors that mediate serum activity to organize the AJC. *Afadin*-KO EpH4 cells transfected with control siRNA (si-Control), LPAR4 siRNA (si-LPAR4), or LPAR5 siRNA (si-LPAR5) were cultured in the serum-containing medium in the presence of DMSO or 10 μM KI16425, an LPAR1/3 inhibitor, for 3 h. The cells were fixed and stained with the anti-ZO-1 Ab and then observed by immunofluorescence microscopy. Quantitative analysis is shown in the *right panel*. The percentages of the ZO-1-positive cells are 84% (si-Control/DMSO, n = 223), 97% (si-LPAR4/DMSO, n = 200), 88% (si-LPAR5/DMSO, n = 218), 92% (si-Control/KI16425 [+], n = 262), 90% (si-LPAR4/KI16425 [+], n = 217), and 45% (si-LPAR5/KI16425 [+], n = 185). Serum (+) indicates the serum-containing medium, whereas serum (−) indicates the serum-free medium; bp indicates base pair; and the scale bars indicate 50 μm. The results are representative of three independent experiments. Ab, antibody; AJC, apical junctional complex; DMSO, dimethyl sulfoxide; LPAR, lysophosphatidic acid receptor.

Six LPARs (LPAR1/2/3/4/5/6) have been identified (34), and of these receptors, all receptors except LPAR3 were expressed in EpH4 cells, as estimated by RT–PCR (Fig. 7*A*). The AJC organization–promoting activity of serum was abolished by the combination of the LPAR1/3 inhibitor KI16425 and LPAR5 siRNA, but not by KI16425 or LPAR5 siRNA alone (Fig. 7*B*). It was not abolished by LPAR4 siRNA or the combinations of KI16425 and LPAR4 siRNA. Essentially the same results were obtained for LPA (Fig. S8). These results indicate that the AJC organization–promoting activity of serum and LPA is mediated by LPAR1/5.

### Involvement of the DAG–nPKC and Rho–ROCK pathway activation in the serum-promoted and LPA-promoted AJC organization

**Figure 8:**
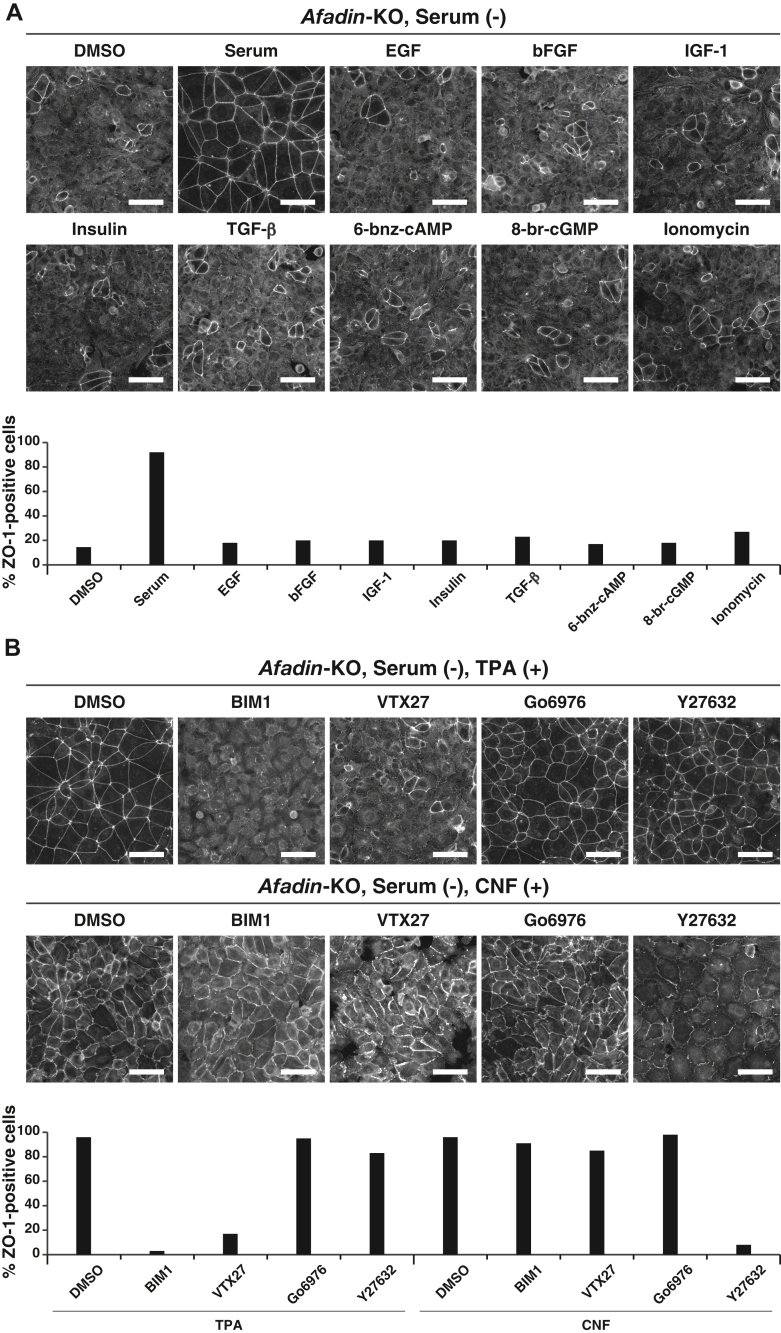
**Restoration of the AJC by the activation of either nPKC or ROCK in *afadin*-KO EpH4 cells cultured in the serum-free medium.***A*, no effect of various extracellular signaling molecules or activators on the AJC maintenance in *afadin*-KO EpH4 cells cultured in the serum-free medium. *Afadin*-KO EpH4 cells were cultured in the serum-free medium in the presence of DMSO, 10% serum, 100 ng/ml EGF, 100 ng/ml bFGF, 100 ng/ml IGF-1, 5 μg/ml insulin, 100 ng/ml TGF-β, 1 mM 6-bnz-cAMP, 1 mM 8-br-cGMP, or 50 nM ionomycin for 8 h. The cells were fixed and stained with the anti-ZO-1 Ab and then observed by immunofluorescence microscopy. Quantitative analysis is shown in the *lower panel*. The percentages of the ZO-1-positive cells are 14% (DMSO, n = 200), 92% (serum, n = 169), 18% (EGF, n = 180), 20% (bFGF, n = 181), 20% (IGF-1, n = 169), 20% (insulin, n = 232), 23% (TGF-β, n = 186), 17% (6-bnz-cAMP, n = 191), 18% (8-br-cGMP, n = 181), and 27% (ionomycin, n = 207). *B*, restoration of the AJC by the activation of either nPKC or ROCK in *afadin*-KO EpH4 cells cultured in the serum-free medium. *Afadin*-KO EpH4 cells were cultured in the serum-free medium in the presence of 50 nM TPA or 2 μg/ml CNF together with DMSO, 5 μM BIM1, 500 nM VTX27, 5 μM Go6976, or 5 μM Y27632 for 8 h. The cells were fixed and stained with the anti-ZO-1 Ab and then observed by immunofluorescence microscopy. Quantitative analysis is shown in the *lower panel*. The percentages of the ZO-1-positive cells are 96% (DMSO/TPA [+], n = 195), 3% (BIM1/TPA [+], n = 194), 17% (VTX27/TPA [+], n = 185), 95% (Go6976/TPA [+], n = 205), 83% (Y27632/TPA [+], n = 189), 96% (DMSO/CNF [+], n = 177), 91% (BIM1/CNF [+], n = 178), 85% (VTX27/CNF [+], n = 180), 98% (Go6976/CNF [+], n = 145), and 8% (Y27632/CNF [+], n = 130). Serum (−) indicates the serum-free medium; and the scale bars indicate 50 μm. The results are representative of three independent experiments. Ab, antibody; AJC, apical junctional complex; bFGF, basic fibroblast growth factor; BIM1, bisindolylmaleimide 1; CNF, cytotoxic necrotizing factor; DMSO, dimethyl sulfoxide; EGF, epidermal growth factor; IGF-1, insulin-like growth factor-1; nPKC, novel PKC; TGF-β, transforming growth factor-β; TPA, 12-*O*-tetradecanoylphorbol 13-acetate.

LPAR1 induces Rho, phospholipase C (PLC), Ras, and PI3K activation and adenylate cyclase inactivation, whereas LPAR5 induces Rho and PLC activation (34). Rho induces ROCK and serum response factor activation, PLC induces the inositol 1,4,5-triphosphate–Ca^2+^ and DAG–PKC pathway activation, Ras induces the Raf-mitogen-activated protein kinase kinase–mitogen-activated protein kinase pathway activation, and PI3K induces Akt and Rac activation (34). We first examined the effect of various extracellular signaling molecules that regulate these signaling pathways on AJC organization in *afadin*-KO EpH4 cells cultured at normal Ca^2+^ in the serum-free medium: these include epidermal growth factor, basic fibroblast growth factor, insulin-like growth factor-1, insulin, and transforming growth factor-β. In addition, we used 6-bnz-cAMP, 8-br-cGMP, ionomycin, the conventional PKC (cPKC)- and nPKC-activating phorbol ester TPA, and the Rho activator cytotoxic necrotizing factor (CNF). Of these molecules examined, TPA and CNF, but not other molecules, substituted for serum and restored the ZO-1 signal at the cell–cell boundaries in *afadin*-KO EpH4 cells cultured at normal Ca^2+^ in the serum-free medium (Fig. 8, *A* and *B*).

PKC comprises a family with nine members that are classified into three groups: cPKC, nPKC, and atypical PKC (40, 41). The TPA-induced restoration of the ZO-1 signal at the cell–cell boundaries in *afadin*-KO EpH4 cells cultured at normal Ca^2+^ in the serum-free medium was abolished by the PKC inhibitors bisindolylmaleimide 1 (BIM1) and VTX27, but not by Go6976 (Fig. 8*B*). BIM1 is an inhibitor of cPKC and nPKC; VTX27 is an inhibitor of nPKC; and Go6976 is an inhibitor of cPKC (42). The CNF-induced restoration of the ZO-1 signal at the cell–cell boundaries in *afadin*-KO EpH4 cells cultured at normal Ca^2+^ in the serum-free medium was abolished by the ROCK inhibitor Y27632 (Fig. 8*B*). The TPA effect was not abolished by Y27632, whereas the CNF effect was not abolished by BIM1, VTX27, or Go6976 (Fig. 8*B*). These results indicate that the nPKC activation is involved in the TPA-promoted AJC organization, whereas the ROCK activation is involved in the CNF-promoted AJC organization, raising the possibility that the DAG–nPKC and Rho–ROCK pathway activation is involved in the serum-promoted AJC organization. It was also noted that TPA restored the reduced cell surface area change shown in *afadin*-KO EpH4 cells cultured in the serum-free medium and that the TPA-induced cell surface area change was abolished by BIM1 and VTX27, indicating that the DAG–nPKC pathway activation has an activity for making the cells flatten out. In contrast, CNF further reduced the cell surface area in *afadin*-KO EpH4 cells cultured in the serum-free medium and the CNF-induced cell surface area change was abolished by Y27632, indicating that the Rho–ROCK pathway activation has an activity for making the cells shrink.

**Figure 9:**
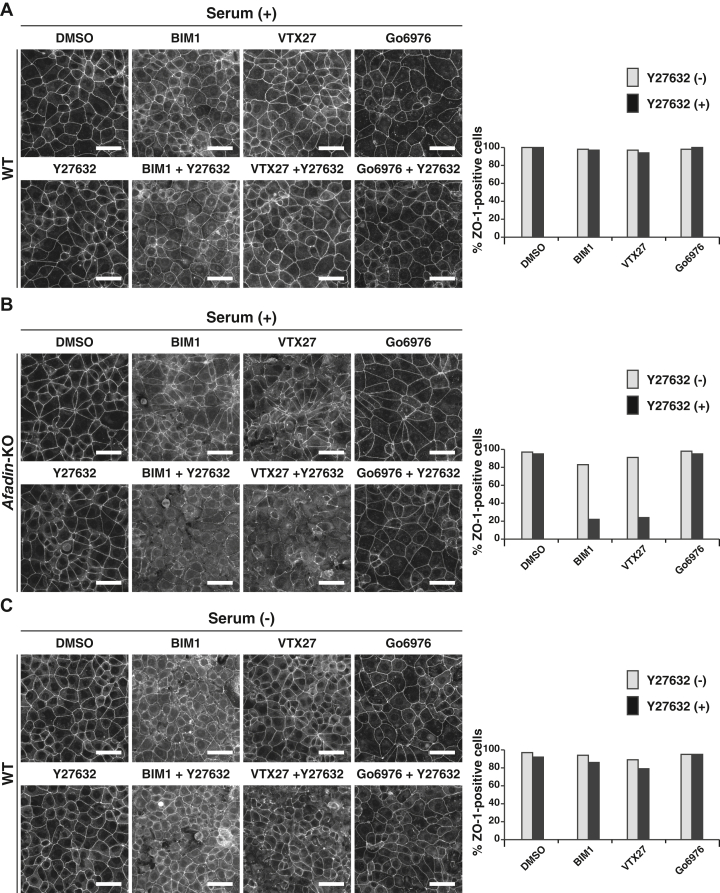
**Requirement of the activation of either nPKC or ROCK for the serum-promoted AJC organization.** WT or *afadin*-KO EpH4 cells were cultured in the serum-containing or serum-free medium in the presence of DMSO or the indicated combinations of inhibitors for 8 h. The cells were fixed and stained with the anti-ZO-1 Ab and then observed by immunofluorescence microscopy. Quantitative analyses are shown in the *right panel*s. The percentages of the ZO-1-positive cells in (*A*) are 100% (DMSO, n = 185), 98% (BIM1, n = 211), 97% (VTX27, n = 203), 98% (Go6976, n = 172), 100% (Y27632, n = 200), 97% (BIM1/Y27632, n = 193), 94% (VTX27/Y27632, n = 194), and 100% (Go6976/Y27632, n = 165). The percentages of the ZO-1-positive cells in (*B*) are 97% (DMSO, n = 197), 83% (BIM1, n = 182), 91% (VTX27, n = 189), 98% (Go6976, n = 166), 95% (Y27632, n = 174), 22% (BIM1/Y27632, n = 170), 24% (VTX27/Y27632, n = 176), and 95% (Go6976/Y27632, n = 165). The percentages of the ZO-1-positive cells in (*C*) are 97% (DMSO, n = 203), 94% (BIM1, n = 190), 89% (VTX27, n = 205), 95% (Go6976, n = 180), 92% (Y27632, n = 192), 86% (BIM1/Y27632, n = 194), 79% (VTX27/Y27632, n = 220), and 95% (Go6976/Y27632, n = 185). Serum (+) indicates the serum-containing medium, whereas serum (−) indicates the serum-free medium; and the scale bars indicate 50 μm. The results are representative of three independent experiments. Ab, antibody; AJC, apical junctional complex; BIM1, bisindolylmaleimide 1; DMSO, dimethyl sulfoxide; nPKC, novel PKC.

We then examined this possibility using these inhibitors of nPKC and ROCK. Any combinations of inhibitors did not disrupt AJC organization in WT EpH4 cells cultured in the serum-containing or serum-free medium (Fig. 9, *A* and *C*). In contrast, the serum-promoted restoration of the ZO-1 signal at the cell–cell boundaries in *afadin*-KO EpH4 cells was abolished by the combination of VTX27 and Y27632 and the combination of BIM1 and Y27632, but not by VTX27 alone, BIM1 alone, or Y27632 alone (Fig. 9*B*). It was not abolished by Go6976 alone or the combination of Go6976 and Y27632 (Fig. 9*B*). It was also noted that BIM1 and VTX27 reduced the cell surface area in *afadin*-KO EpH4 cells cultured in the serum-containing medium, indicating that the serum activity for making the cells flatten out is mediated by the DAG–nPKC pathway activation. Essentially the same results were obtained for the LPA-promoted restoration of the ZO-1 signal at the cell–cell boundaries in *afadin*-KO EpH4 cells (Fig. S9). These results indicate that the activation of both nPKC and ROCK is involved in the serum-promoted and LPA-promoted AJC organization in a mutually independent, but complementary, manner.

### The Rho–ROCK pathway activation–mediated AJC organization in a manner independent of myosin II-induced actomyosin contraction

**Figure 10:**
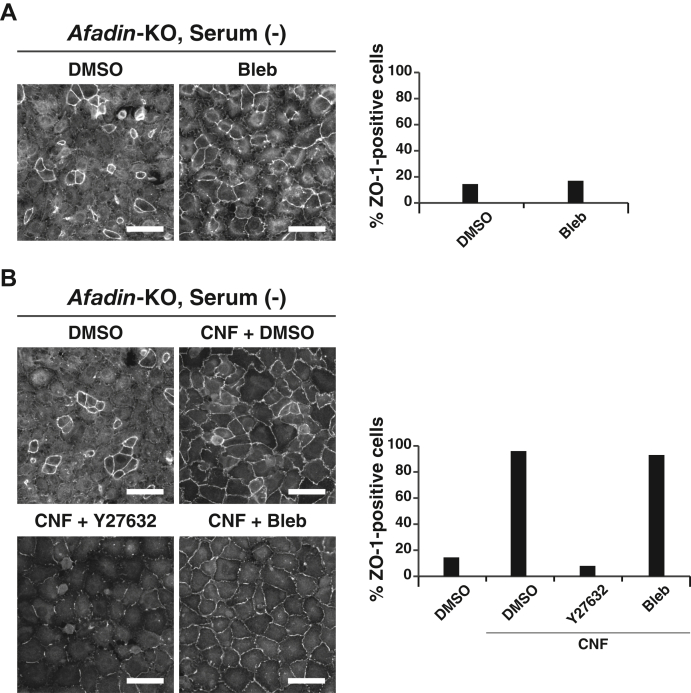
**No remarkable effect of myosin II activation on the AJC disruption or the Rho–ROCK pathway-mediated AJC organization.***A*, *Afadin*-KO EpH4 cells were cultured in the serum-free medium in the presence of DMSO or 25 μM blebbistatin for 8 h. The cells were fixed and stained with the anti-ZO-1 Ab and then observed by immunofluorescence microscopy. Quantitative analysis is shown in the *right panel*. The percentages of the ZO-1-positive cells are 14% (DMSO, n = 200) and 17% (Bleb, n = 180). *B*, *Afadin*-KO EpH4 cells were cultured in the serum-free medium or the serum-free medium supplemented with 2 μg/ml CNF together with DMSO, 5 μM Y27632, or 25 μM blebbistatin for 8 h. The cells were fixed and stained with the anti-ZO-1 Ab and then observed by immunofluorescence microscopy. Quantitative analysis is shown in the *right panel*. The percentages of the ZO-1-positive cells are 14% (DMSO, n = 200), 96% (DMSO/CNF [+], n = 177), 8% (Y27632/CNF [+], n = 130), and 93% (Bleb/CNF [+], n = 136). Serum (−) indicates the serum-free medium; Bleb indicates blebbistatin; and scale bars indicate 50 μm. The results are representative of three independent experiments. Ab, antibody; AJC, apical junctional complex; CNF, cytotoxic necrotizing factor; DMSO, dimethyl sulfoxide.

To understand how the serum-induced nPKC and ROCK activation promotes AJC organization, we tested the possible involvement of myosin II activation in AJC organization, because the AJC is disrupted by the reduced cell adhesion activities of CAMs, the excess contraction of the circumferential F-actin structure that pulls the plasma membrane inward to dissociate the CAM-mediated adhesion, or both (43, 44). The contraction of the circumferential F-actin structure is induced by myosin II ATPase activation, which is regulated by many signaling molecules, including CaMKII, ROCK, PKC, PKG, and PKA, through myosin light chain kinase and phosphatase (45). We examined, using the myosin II ATPase inhibitor blebbistatin, whether the excess contraction of the circumferential F-actin structure is involved in AJC disorganization observed in *afadin*-KO EpH4 cells cultured in the serum-free medium. Blebbistatin hardly restored the ZO-1 signal at the cell–cell boundaries in *afadin*-KO EpH4 cells cultured at normal Ca^2+^ in the serum-free medium (Fig. 10*A*). CNF decreased the cell surface area of *afadin*-KO EpH4 cells cultured at normal Ca^2+^ in the serum-free medium, and these changes were abolished by either Y27632 or blebbistatin, probably because of the inhibition of the excess contraction of the circumferential F-actin structure, making sure that blebbistatin as well as Y27632 functions properly (Fig. 10*B*). However, the CNF-induced restoration of the ZO-1 signal at the cell–cell boundaries in *afadin*-KO EpH4 cells cultured at normal Ca^2+^ in the serum-free medium was abolished by Y27632, but not by blebbistatin (Figs. 10*B* and 8*B*). These results indicate that myosin II activation is hardly involved in AJC disorganization and that the Rho–ROCK pathway activation mediates the serum-promoted AJC organization in a myosin II activity–independent manner, although it has been shown that the Rho–ROCK pathway activation induces the contraction of the circumferential F-actin structure through the myosin II ATPase activation (43, 46).

## Discussion

**Figure 11:**
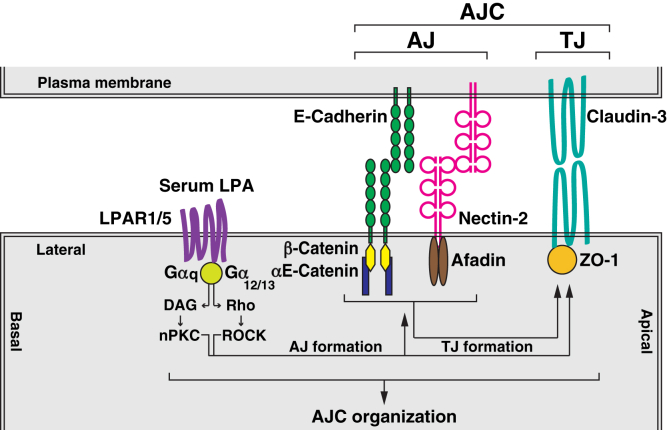
**Schematic diagram of the regulatory mechanism for the serum LPA-promoted AJC organization.** Major AJC components are shown. Major AJ CAMs, E-cadherin and nectin-2, bind the β-catenin−αE-catenin complex and afadin, respectively. Major TJ CAM claudin-3 binds ZO-1. The present study revealed that serum LPA has an AJC organization–promoting activity, and that serum LPA has a TJ formation–promoting activity in a manner independent of the AJ components. These serum LPA activities are mediated through LPAR1/5 *via* the DAG–nPKC and Rho–ROCK pathway activation. AJ, adherens junction; AJC, apical junctional complex; CAM, cell adhesion molecule; DAG, diacylglycerol; LPA, lysophosphatidic acid; LPAR, lysophosphatidic acid receptor; nPKC, novel PKC; TJ, tight junction.

Our series of previous studies using mainly cultured MDCK cells revealed that nectin-based cell adhesion initiates AJ formation and then TJ formation, resulting in AJC organization, in an afadin-dependent manner (8), but the role of serum in these processes remained unknown. We investigated here the role of serum in these processes under various conditions and revealed for the first time that serum showed a novel activity to promote AJC organization, as schematically shown in Figure 11. We first showed here that in WT EpH4 cells cultured at normal Ca^2+^, serum promoted AJC organization. It is currently under investigation whether the AJC-promoting activity of serum is also observed in MDCK cells.

We then attempted here to identify the individual factor(s) responsible for these functions from serum in the medium and identified this molecule as LPA. The concentration of LPA in serum is frequently increased in inflammation, development, and progression of various cancers (35). These previous results suggested an AJC organization–disrupting activity of LPA. It was recently shown that LPA enhances the intestinal epithelial barrier function and susceptibility to dextran sodium sulfate–induced colitis through LPAR1 in mouse colon (47); that LPA attenuates radiation-induced disruption of the AJC and mucosal barrier through LPAR2 in mouse colon (48); and that LPA induces the clustering of neuroblastoma cells overexpressing LPAR4 (49). However, in these previous studies, the AJC organization–promoting activity of LPA was not systematically studied. It was also shown that LPA enhances vascular endothelial (VE)-cadherin-based AJ formation in VE cells (50), but in this study, the accumulation of VE-cadherin at the cell–cell boundaries, only a part of the process of AJC organization, was shown in VE cells and a TJ formation–promoting or AJC organization–promoting activity of LPA was not shown. In contrast, we clearly revealed here an AJC organization–promoting activity of LPA. We showed here that LPA promoted these processes through both LPAR1/5 *via* the DAG–nPKC and Rho–ROCK pathway activation in a mutually independent, but complementary, manner (Fig. 11). Rho is likely to be activated by p115 RhoGEF associated with LPAR1/5 *via* Gα12/13, whereas DAG is likely to be produced by PLC associated with LPAR1/5 *via* Gαq (34, 51). However, it remained unknown how LPA shows either an AJC organization–disrupting or an AJC organization–promoting activity depending on cell types.

We previously showed in MDCK cells that disruption of F-actin structures by cytochalasin or latrunculin A impairs AJC organization (52) and that nectin induces Cdc42 and Rac activation, which reorganizes F-actin structures, although this nectin-induced activation of Cdc42 and Rac is transient (53). These results suggested that the F-actin structures reorganized by Cdc42 and Rac are involved in AJC organization at least at their early stages. Taken together with these previous observations, the present results suggest that serum LPA reorganizes the F-actin structures prereorganized by the nectin-based cell adhesion through the DAG–nPKC and Rho–ROCK pathway activation, eventually promoting AJC organization. The mesh-like and circumferential F-actin structures are associated with the plasma membrane at the AJC, particularly at the AJs, but it remains elusive how LPA reorganizes these F-actin structures through the DAG–nPKC and Rho–ROCK pathway activation to promote AJC organization.

The association of the mesh-like and circumferential F-actin with the plasma membrane at the AJC and their dissociation from there are balanced by the association strength of these two types of F-actin structures with the plasma membrane. It was previously shown that the Rho–ROCK pathway activation induces the contraction of the circumferential F-actin structure through the myosin II ATPase activation (43, 46), but we showed here that the Rho–ROCK–myosin II pathway activation was too small to contract the circumferential F-actin structure to dissociate the mesh-like F-actin structure from the plasma membrane, eventually leading to AJC disorganization. Therefore, the force of the association of the mesh-like F-actin structure with the plasma membrane may be stronger than that of the dissociation of the mesh-like F-actin structure from the plasma membrane by the Rho–ROCK–myosin II pathway activation–mediated circumferential F-actin contraction. Thus, it should be emphasized here that the Rho–ROCK-induced myosin II activation is hardly involved in AJC disorganization. It remains elusive how the LPA-induced Rho–ROCK pathway activation regulates AJC organization, but not the myosin II pathway activation–mediated circumferential F-actin contraction. The exact mechanisms for these different roles of the Rho–ROCK pathways are not clear, but LPA may induce the p115 RhoGEF activation through LPAR1/5, resulting in the activation of only a subset of the Rho–ROCK pathway localized just beneath the plasma membrane and the mesh-like F-actin, which might favor the regulation of the association of this F-actin structure with the plasma membrane rather than the myosin II pathway activation–mediated circumferential F-actin contraction.

In the processes of AJC organization, TJ formation is generally dependent on AJ formation (7), and E-cadherin-based cell adhesion had been shown to promote TJ formation in a β-catenin–αE-catenin complex-dependent manner (9, 36). Here, we showed that E-cadherin-based cell adhesion is not required for TJ formation in WT EpH4 cells cultured at low Ca^2+^ in the serum-containing medium and in *E-cadherin*-KO and *αE-catenin-*KO EpH4 cells cultured at normal Ca^2+^ in the serum-containing medium. Under these conditions, LPA promoted TJ formation in a manner independent of E-cadherin-based cell adhesion through LPAR1/5 *via* the DAG–nPKC and Rho–ROCK pathway activation in a mutually independent, but complementary, manner. These results were consistent with the earlier observations that under several artificial conditions, the TJs are formed without E-cadherin-based cell adhesion (1): TPA induces TJ-like structure formation without E-cadherin-based cell adhesion in MDCK cells cultured at low Ca^2+^ in the serum-free medium (54). This TPA-induced TJ-like structure formation is dependent on nectin-1-based cell adhesion, but not on E-cadherin-based cell adhesion, in MDCK cells cultured at low Ca^2+^ in the serum-free medium (29). Under these conditions, non-*trans*-interacting E-cadherin and its associating β-catenin and αE-catenin are recruited to the nectin-1-based cell adhesion sites (29). PKC is involved in this TPA-induced TJ-like structure formation, although the member of the PKC family involved in this TPA action remained unidentified (29); in cadherin-deficient L fibroblasts stably expressing both exogenous JAM and nectin-1, nectin-1-based cell adhesion recruits JAM-based cell adhesion in a manner independent of cadherin-based cell adhesion (24); in a cellular system with which epithelial-like TJs and AJs are reconstituted in fibroblasts, JAM-based cell adhesion is formed in a manner dependent on nectin-3-based cell–cell adhesion, but not on E-cadherin-based cell adhesion (55); and TJs are formed in a manner dependent on nectin-based cell adhesion, but not on E-cadherin-based cell adhesion, in annexin II-knockdown MDCK cells (56). In these previous and present studies, however, it remained unknown whether the structurally and functionally genuine TJs are formed or whether the TJs are formed at the apical side of the AJs. In the cellular system in which the TJs and the AJs are reconstituted in fibroblasts, the TJs are formed at the basal side of the AJs, but this inversed alignment is reversed by the expression of the cell polarity proteins, including Par-3, atypical PKC, Par-6, Crb3, Pals1, and Patj (55). Collectively, these previous and present results indicate that the AJC component and cell polarity molecules are involved in AJC organization and epithelial apicobasal polarity formation in which the TJs are formed at the apical side of the AJs but suggest that LPA is not involved in these AJ, TJ, or AJC structure formation or this apicobasal polarity formation but promotes these processes through LPAR1/5 *via* the DAG–nPKC and Rho–ROCK pathway activation, although it remains unknown how these pathways regulate these processes.

The physiological and/or pathological relevance/significance of the AJC organization–promoting activity of LPA are important because we used the Ca^2+^ switch assay, *E-cadherin*-KO, *αE-catenin-KO*, or *afadin*-KO EpH4 cells. Extracellular Ca^2+^ concentrations are not presumed to be decreased to micromolar levels under any physiological or pathological conditions, but we showed here that at physiological millimolar level of Ca^2+^ concentrations, LPA promoted AJC organization. Because the concentration of LPA in serum is changed under various physiological and/or pathological conditions (35), the AJC organization–promoting activity of LPA is likely physiologically and/or pathologically relevant and significant. The concentration of LPA in serum is approximately 5 to 10 μM, whereas that of plasma is approximately 0.1 to 1 μM LPA (35, 57). We showed here that 2% serum was sufficiently effective for AJC organization. Although we did not examine whether plasma also has the AJC organization–promoting activity, it is probable that it does, considering that the concentration of LPA in 2% serum is comparable to that in the plasma. We showed here that 10 μM LPA was sufficiently effective for AJC organization but did not examine whether the lower concentration of LPA, which is comparable to that in 2% serum or plasma, is sufficiently effective for AJC organization. If this is not the case, serum might contain a cofactor(s) of LPA that enhances the AJC organization–promoting activity of LPA and the unidentified cofactor(s) of LPA may play an important role in AJC organization under physiological or pathological conditions. On the other hand, it was previously reported that approximately 20 μM LPA was detected in ascites derived from ovarian cancer patients (58). Therefore, cells may be exposed to 20 μM LPA, where LPA alone is sufficiently effective for AJC organization, under certain pathological conditions. We are currently investigating these issues.

As for the TJ formation–promoting activity of LPA in an E-cadherin-based cell adhesion–independent manner in *E-cadherin*-KO and *αE-catenin-*KO and the AJC organization–promoting activity of LPA in *afadin*-KO EpH4 cells, these activities may operate in various cancers in which E-cadherin, αE-catenin, or afadin is lost or downregulated (59, 60, 61). In addition, the reduced activities of E-cadherin, αE-catenin, and afadin have been reported: For instance, inactivating mutations of E-cadherin are frequently detected in infiltrating lobular breast carcinomas and in diffuse gastric cancer (62). E-cadherin phosphorylated at threonine 790 reduces β-catenin binding and suppresses the function of E-cadherin (63). Afadin is phosphorylated at serine 1718 by Akt, which is activated in response to insulin-like growth factor-1, or by oncogenic alterations in the phosphatase and tensin homolog–PI3K–Akt pathway, and translocated into the nucleus, disrupting the AJs and enhancing migration in breast cancers (64). It was not examined whether afadin is downregulated in the radiation-induced AJC disorganization in the mouse colon (48) or the dextran sodium sulfate–induced colitis in the mouse colon (47); however, afadin may be downregulated, and serum LPA may at least partly complement afadin and promote AJC organization. In the vascular network formation, afadin is localized at the leading edges of moving endothelial cells (65) and may not be present in an amount sufficient for AJC organization, but LPA may at least partly complement afadin and promote AJC organization. Therefore, these present and previous results collectively indicate that the AJC organization–promoting activity of LPA may play a role in maintaining the structures and functions of these cell adhesion apparatuses in cells in which E-cadherin, αE-catenin, or afadin does not play these roles sufficiently.

## Experimental procedures

### Cell culture

WT EpH4 cells and *afadin*-KO EpH4 cells were described previously (27). MDCK cells were described previously (20). The cells were maintained in Dulbecco's modified Eagle's medium (DMEM) with 10% FBS and 1% penicillin/streptomycin and cultured under 5% CO_2_ at 37 °C. Lipofectamine 3000 (Thermo Fisher Scientific) was used as a reagent for plasmid transfection.

### Generation of *E-cadherin*-KO or *αE-catenin*-KO EpH4 cells

WT EpH4 cells cultured in DMEM with 10% FBS and 1% penicillin/streptomycin were transfected with target-specific CRISPR/Cas9 KO Plasmids targeting for *E-cadherin* or *αE-catenin* gene (Santa Cruz Biotechnology), which contained the GFP gene cassette as a reporter, and cultured for 16 h. After single GFP-positive cell was sorted into 96-well plates using BD FACSAria cell-sorting system (BD Biosciences), the cells were cultured for 2 weeks in DMEM/F12 with 2% FBS, 5 μg/ml insulin, and 50 μg/ml gentamicin, where the fresh culture medium was mixed to the conditioned medium from WT EpH4 cells. Single cell–derived clones were then transferred into a single well in 24-well culture dishes and cultured in DMEM/F12 with 2% FBS, 5 μg/ml insulin, and 50 μg/ml gentamicin. Then, the clones of interest in which the *E-cadherin* or *αE-catenin* gene was knocked out were screened by Western blotting. The positive clones were cultured for maintenance under normal conditions.

### Abs and reagents

The Abs and reagents used in this study are listed in Tables S1 and S2, respectively. A rat anti-E-cadherin Ab (ECCD2) was a kind gift from Dr Masatoshi Takeichi.

### Ca^2+^ switch assay

Cells to be examined were seeded in a 24-well plate (1.5 × 10^5^ cells per well) and cultured under normal conditions for 20 h. The cells were precultured at 5 mM EGTA in DMEM without FBS for 3 h and washed twice with the same culture medium. Then, the cells were recultured either in the presence or the absence of 5 mM EGTA in DMEM either with or without FBS for various periods and washed twice with the same culture media. Then, the cells were subjected to immunocytochemistry.

### Immunocytochemistry

The cells to be examined were seeded in a 24-well plate (1.4 × 10^5^ cells per well) and cultured under normal conditions for 20 h until cells just reached 100% confluence. After subjecting the cells to various treatments, they were fixed with a fixative containing 2% paraformaldehyde, 4% sucrose, 1 mM sodium pyruvate, Hanks’ balanced salt solution containing 1 mM CaCl_2_ and 1 mM MgCl_2_ (Thermo Fisher Scientific), and 10 mM Hepes (pH 7.4) at 37 °C for 15 min. The fixed cells were permeabilized with 0.25% Triton X-100 in PBS for 10 min and then blocked in PBS containing 10% normal goat serum at room temperature for 20 min. The cells were then incubated with primary Abs in PBS containing 20% Block Ace (KAC) at 4 °C overnight. After washed three times with PBS at room temperature, the cells were incubated with Alexa Fluor–conjugated secondary Abs (Thermo Fisher Scientific) at room temperature for 45 min and then washed three times with PBS. The samples were then mounted in a FluorSave reagent (Merck Millipore). The images were acquired using a Nikon C2 confocal system (Nikon, Inc) with a Plan Apo 60×/1.2 numerical aperture water immersion objective lens (Nikon, Inc). Maximum signal intensity projection images were obtained using the ImageJ software program (National Institutes of Health and the Laboratory for Optical and Computational Instrumentation).

### Quantification of percent of E-cadherin-positive cells, percent of nectin-2-positive cells, and percent of ZO-1-positive cells

The intensities of the signals for each AJC component were normalized, where the average intensities of the signals for each AJC component in WT EpH4 cells cultured at normal Ca^2+^ in the serum-containing medium were defined as 1.0. The signals for each AJC component were defined to be positive when the normalized intensity was more than 0.5. In the Ca^2+^ switch assay and the serum depletion assay using *E-cadherin*-KO EpH4 cells and *αE-catenin-KO* EpH4 cells, the percentages of the cells at least one side of which was positive for the signals were calculated. In the serum depletion assay using *afadin*-KO EpH4 cells, the percentages of the cells at all sides of which were positive for the signals were calculated.

### Immunoprecipitation assay

The cells were washed once, scraped from the dishes in ice-cold PBS, and lysed in a lysis buffer (20 mM Tris–HCl at pH 7.5, 1% Triton X-100, 10% glycerol, 150 mM NaCl, 1 mM EGTA, 1 mM EDTA, 1 mM NaF, and 1 mM dithiothreitol). The cell lysates were obtained by centrifugation at 20,000*g* for 5 min. The cell lysates were incubated with the rabbit anti-αΕ-catenin Ab-conjugated protein A-Sepharose (GE Healthcare) at 4 °C for 90 min. After the beads were washed extensively with the lysis buffer, proteins bound to the beads were eluted using an SDS sample buffer (60 mM Tris–HCl at pH 6.7, 3% SDS, 2% 2-mercaptoethanol, and 5% glycerol) and boiled for 5 min. The samples were separated by SDS-PAGE, followed by Western blotting with the indicated Abs.

### Western blotting

Samples separated by SDS-PAGE were transferred to polyvinylidene difluoride membranes (Merck Millipore). After the membranes were blocked with 5% skim milk in Tris-buffered saline, they were incubated with the indicated Abs. After the membranes were washed with Tris-buffered saline containing 0.05% Tween-20 three times, they were incubated with horseradish peroxidase–conjugated anti-rabbit or anti-rat immunoglobulin G Abs. Protein signals were detected using Western Blotting Substrate Plus (Thermo Fisher Scientific).

### Serum lipid extraction and separation

For 800 μl of FBS, total lipids were extracted with 5 ml of chloroform/methanol/1 N HCl in H_2_O (2:2:1, by volume), according to the Bligh and Dyer method (38). The organic phase was concentrated by evaporation with nitrogen blowdown. The aqueous phase was concentrated by evaporation under reduced pressure. Then, half of the organic phase was reconstituted with 1 ml of methanol and subjected to lipid separation using a solid-phase C18 cartridge as described previously (66). Briefly, an ISOLUTE C18 column (500 mg; Biotage) was equilibrated with 3 ml of methanol and 6 ml of H_2_O. Nine milliliters of H_2_O was added to the samples and rapidly loaded onto the conditioned column, followed by elution with 9 ml of hexane (neutral lipid fraction), 9 ml of methyl formate (fatty acid fraction), and 9 ml of methanol (phospholipid/lysophospholipid/glycolipid fraction). Each fraction was dried with nitrogen blowdown, reconstituted with 400 μl of DMEM supplemented with 1% bovine serum albumin without serum, and added to the cells so that the final concentration of each fraction was 20 v/v%.

### CIAP treatment of serum

For 30 μl of FBS, either 1 μl of CIAP (Takara Bio) or 1 μl of a buffer containing 10 mM Tris–HCl (pH 8.0), 50 mM KCl, 1 mM MgCl_2_, and 50% glycerol as a negative control was mixed and incubated at 37 °C for 24 h. Then, the reaction solution was subjected to heat treatment at 56 °C for 1 h and added to the cells so that the final concentration was 2 v/v%.

### RT–PCR

The cells were washed with ice-cold PBS once and lysed with TRIzol (Thermo Fisher Scientific). Cell lysates were obtained by centrifugation at 20,000*g* for 5 min. After mixed with 1/5 volume of chloroform, the aqueous phase fraction was obtained by centrifugation at 20,000*g* for 5 min. Total RNAs were extracted from the aqueous phase fraction with RNeasy Mini Kit (Qiagen) in accordance with the manufacturer’s instructions. To prepare the complementary DNAs, 5 μg of the total RNAs was subjected to reverse transcription in a 20 μl reaction each using SuperScript IV reverse transcriptase (Thermo Fisher Scientific). Then, the reaction solution (1 μl for each sample) was subjected to PCR in a 10 μl reaction using GoTaq DNA Polymerase (Promega) for 30 cycles. The sequences of the primers against mouse LPARs, β-actin, and GAPDH are listed in Table S3.

### Knockdown experiments

*Afadin*-KO EpH4 cells were seeded in a 24-well plate (0.3 × 10^5^ cells per well) and cultured under normal conditions for 20 h. Prior to the transfection of the siRNAs, the medium was replaced with DMEM/F12 without FBS. To prepare the lipid–siRNA complex, the siRNAs (5 pmol for each well) and Lipofectamine RNAiMAX reagent (1.5 μl for each well; Thermo Fisher Scientific) were incubated in the Opti-MEM (50 μl for each well) at room temperature for 5 min. Then, the lipid–siRNA complex was added to the cells. After incubation of the cells for 72 h, the medium was replaced with DMEM with 2% FBS and incubated for 3 h. Then, the cells were used for experiments. The sequences of the siRNAs against mouse LPAR4/5 and a negative-control siRNA are listed in Table S4.

## Data availability

All data generated or analyzed during this study are included in this article and its supporting information files.

## Supporting information

This article contains supporting information. Table S1 shows the Abs used in this study. Table S2 shows the reagents used in this study. Table S3 shows the sequences of the primers against mouse LPARs, β-actin, and GAPDH. Table S4 shows the sequences of the siRNAs against mouse LPAR4/5 and a negative-control siRNA.

## Conflict of interest

The authors declare that they have no conflicts of interest with the contents of this article.
