# Supplementary material for: Identification of lysophosphatidic acid in serum as a factor that promotes epithelial apical junctional complex organization
Source: J Biol Chem. 2022 Aug 27;298(10):102426. doi: 10.1016/j.jbc.2022.102426 (PMC9520027; doi:10.1016/j.jbc.2022.102426)
Supplement: Supplementary information [file mmc1.pdf]

Identification of lysophosphatidic acid in serum as a factor that promotes epithelial apical junctional complex organization

Shotaro Sakakibara[1,2], Ayuko Sakane[1,3]*, Takuya Sasaki[1], Masakazu Shinohara[4,5], Tomohiko Maruo[1,2], Muneaki Miyata[2], Kiyohito Mizutani[2]*, and Yoshimi Takai[2]*

[1]Department of Biochemistry, Tokushima University Graduate School of Medicine, Tokushima 770-8503, Japan

[2]Division of Pathogenetic Signaling, Department of Biochemistry and Molecular Biology, Kobe University Graduate School of Medicine, Kobe 650-0047, Japan

[3]Department of Interdisciplinary Researches for Medicine and Photonics, Institute of Post-LED Photonics, Tokushima University, Tokushima 770-8503, Japan

[4]Division of Epidemiology, Kobe University Graduate School of Medicine, Kobe, 650-0017, Japan

[5]The Integrated Center for Mass Spectrometry, Kobe University Graduate School of Medicine, Kobe, 650-0017, Japan

*Correspondence: ytakai@med.kobe-u.ac.jp (YT), mizutani@med.kobe-u.ac.jp (KM), or sakane@tokushima-u.ac.jp (AS)

Present address: Tomohiko Maruo, Department of Molecular and Cellular Neurobiology, Kitasato University Graduate School of Medical Sciences, Department of Biochemistry, Kitasato University School of Medicine, Sagamihara, 252-0374, Japan

Running title: AJC organization-promoting activity of LPA

Keywords: apical junctional complex, adherens junctions, tight junctions, cadherin, catenin, afadin, LPA

Figure S1: AJC organization-promoting activity of serum and LPA in WT EpH4 cells.

Figure S2: TJ formation-promoting activity of serum and LPA in *E-cadherin*-KO EpH4 cells.

Figure S3: TJ formation-promoting activity of serum and LPA in *αE-catenin*-KO EpH4 cells.

Figure S4: AJC organization-promoting activity of serum in a manner that is independent of and mostly complementary to afadin.

Figure S5: Apparently normal AJC organization in a few *afadin*-KO cells cultured in the serum-free medium.

Figure S6: No requirement of afadin or serum for the formation of the αE-catenin–β-catenin–E-cadherin complex at AJs.

Figure S7: Time-dependent AJC restoration by LPA re-addition in *afadin*-KO EpH4 cells precultured in the serum-free medium.

Figure S8: Involvement of LPAR in the LPA-promoted AJC organization.

Figure S9: Involvement of nPKC and ROCK activation in the LPA-promoted AJC organization.

Table S1: Antibodies used in this study.

Table S2: Reagents used in this study.

Table S3: Sequences of the primers against mouse LPARs, β-actin, and GAPDH.

Table S4: Sequences of the siRNAs against mouse LPAR4/5 and a negative control siRNA.

Fig. S1

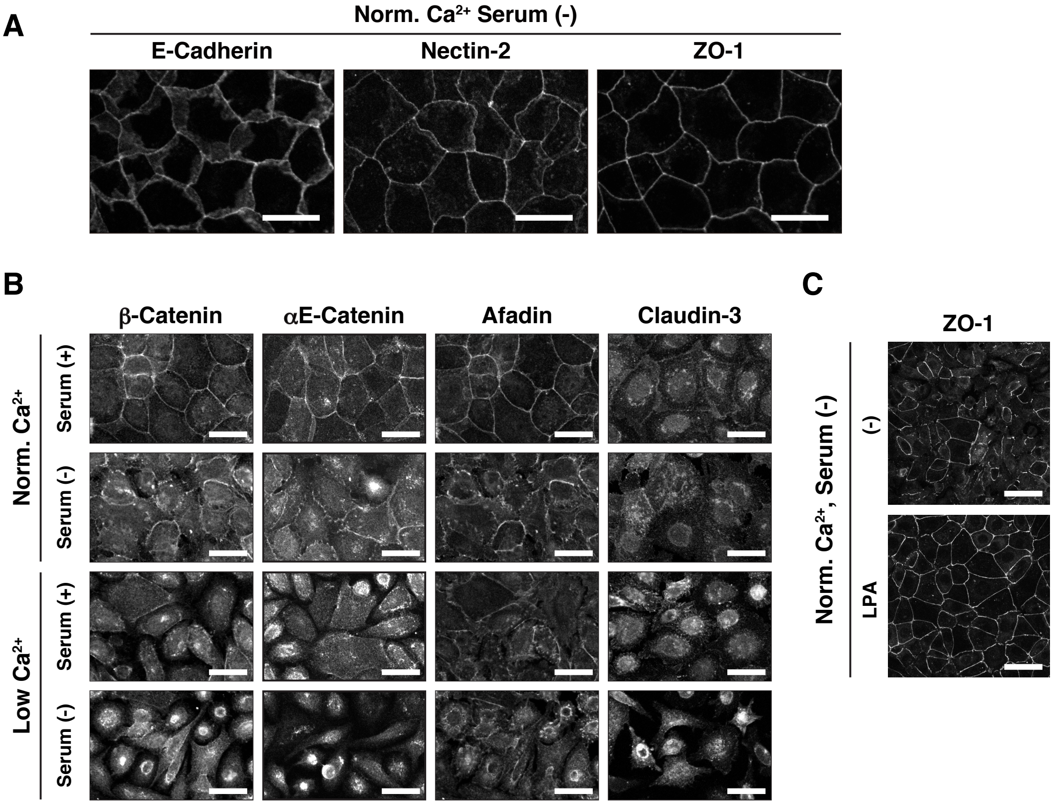

Figure S1. AJC organization-promoting activity of serum and LPA in WT EpH4 cells. (A) AJC restoration 24 h after Ca^{2+} switch in the serum-free medium. WT EpH4 cells pre-cultured at 5 mM EGTA in the serum-free medium for 3 h were re-cultured at 5 mM EGTA in the serum-free medium for 24 h. The cells were fixed and stained with the indicated Abs, and then observed by immunofluorescence microscopy. (B) AJC organization-promoting activity of serum. WT EpH4 cells pre-cultured at 5 mM EGTA in the serum-free medium for 3 h were re-cultured either in the presence or absence of 5 mM EGTA in the serum-containing or serum-free medium for 2 h. The cells were fixed and stained with the indicated Abs, and then observed by immunofluorescence microscopy. (C) AJC organization-promoting activity of LPA. WT EpH4 cells pre-

cultured at 5 mM EGTA in the serum-free medium for 3 h were re-cultured in the serum-free medium either in the presence or absence of 10 μM LPA for 2 h. The cells were fixed and stained with the anti-ZO-1 Ab, and then observed by immunofluorescence microscopy. Serum (+) indicates the serum-containing medium whereas Serum (-) indicates the serum-free medium; Norm. Ca^{2+} indicates re-culturing the cells in the absence of EGTA whereas Low Ca^{2+} indicates re-culturing the cells in the presence of 5 mM EGTA, respectively; and scale bars indicate 25 μm (A) and 50 μm (B). The results are representative of three independent experiments.

Fig. S2

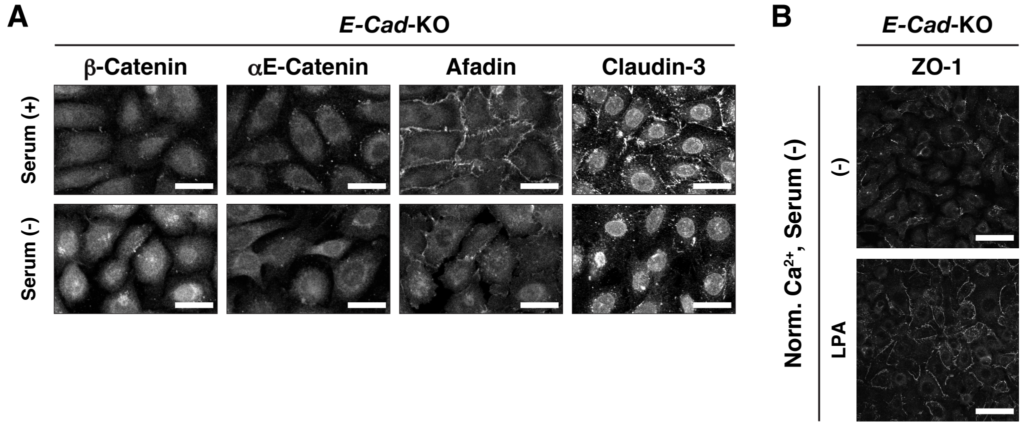

Figure S2. TJ formation-promoting activity of serum and LPA in *E-cadherin*-KO EpH4 cells. (A) Activity of serum for the localization of AJC components in *E-cadherin*-KO EpH4 cells. *E-Cadherin*-KO EpH4 cells were cultured in the serum-containing or serum-free medium for 2 h. The cells were fixed and stained with the indicated Abs, and then observed by immunofluorescence microscopy. (B) Activity of LPA for TJ formation in *E-cadherin*-KO EpH4 cells. *E-Cadherin*-KO EpH4 cells were cultured in the serum-free medium either in the presence or absence of 10 μM LPA for 2 h. The cells were fixed and stained with the anti-ZO-1 Ab, and then observed by immunofluorescence microscopy. Serum (+) indicates the serum-containing medium whereas Serum (-) indicates the serum-free medium; E-Cad indicates E-cadherin; and scale bars indicate 25 μm (A) and 50 μm (B). The results are representative of three independent experiments.

Fig. S3

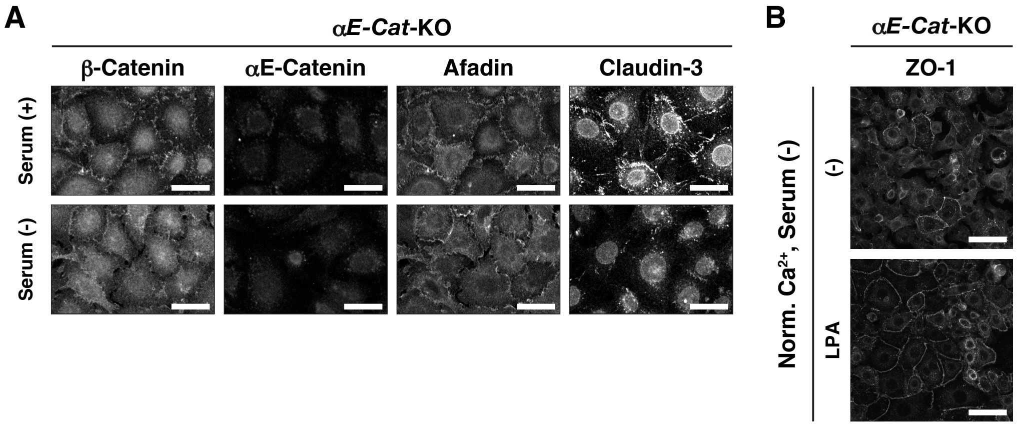

Figure S3. TJ formation-promoting activity of serum and LPA in *αE-catenin*-KO EpH4 cells. (A) Activity of serum for the localization of AJC components in *αE-catenin*-KO EpH4 cells. *αE-Catenin*-KO EpH4 cells were cultured in the serum-containing or serum-free medium for 2 h. The cells were fixed and stained with the indicated Abs, and then observed by immunofluorescence microscopy. (B) Activity of LPA for TJ formation in *αE-catenin*-KO EpH4 cells. *αE-catenin*-KO EpH4 cells were cultured in the serum-free medium either in the presence or absence of 10 μM LPA for 2 h. The cells were fixed and stained with the anti-ZO-1 Ab, and then observed by immunofluorescence microscopy. Serum (+) indicates the serum-containing medium whereas Serum (-) indicates the serum-free medium; αE-Cat indicates αE-catenin; and scale bars indicate 25 μm (A) and 50 μm (B). The results are representative of three independent experiments.

Fig. S4

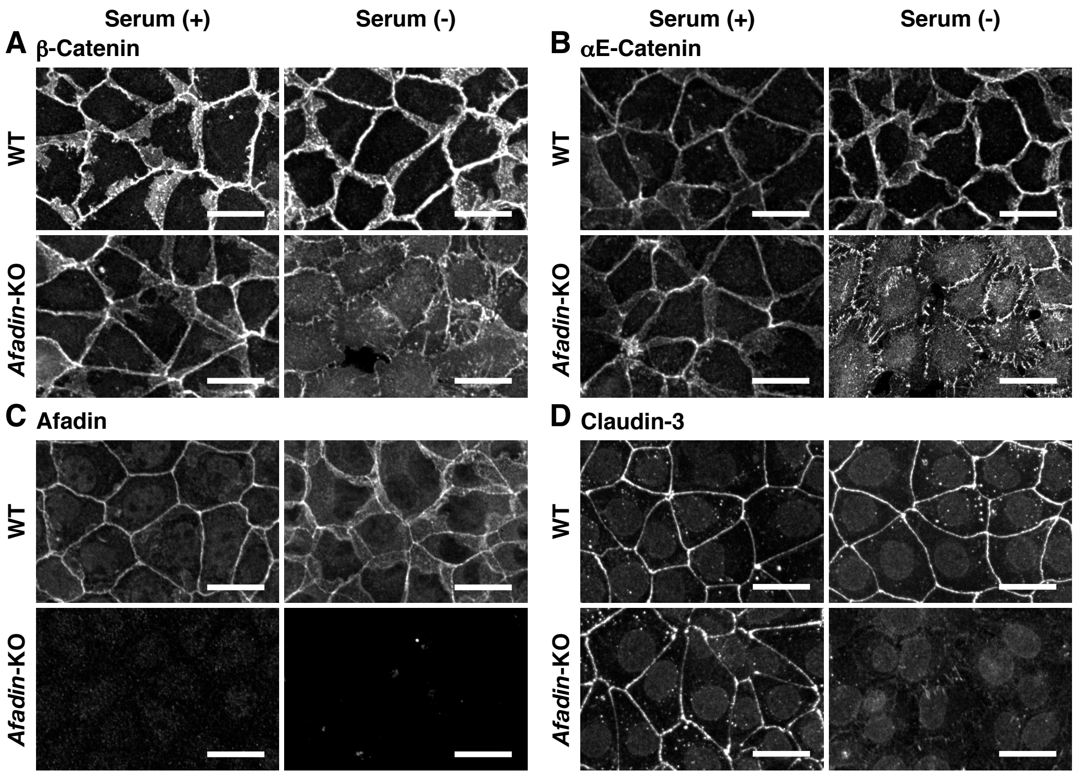

Figure S4. AJC organization-promoting activity of serum in a manner that is independent of and mostly complementary to afadin. WT or *afadin*-KO cells were cultured in the serum-containing or serum-free medium for 8 h. The cells were fixed and stained with the indicated Abs, and then observed by immunofluorescence microscopy. Serum (+) indicates the serum-containing medium whereas Serum (-) indicates the serum-free medium: and scale bars indicate 25 μm. The results are representative of three independent experiments.

Fig. S5

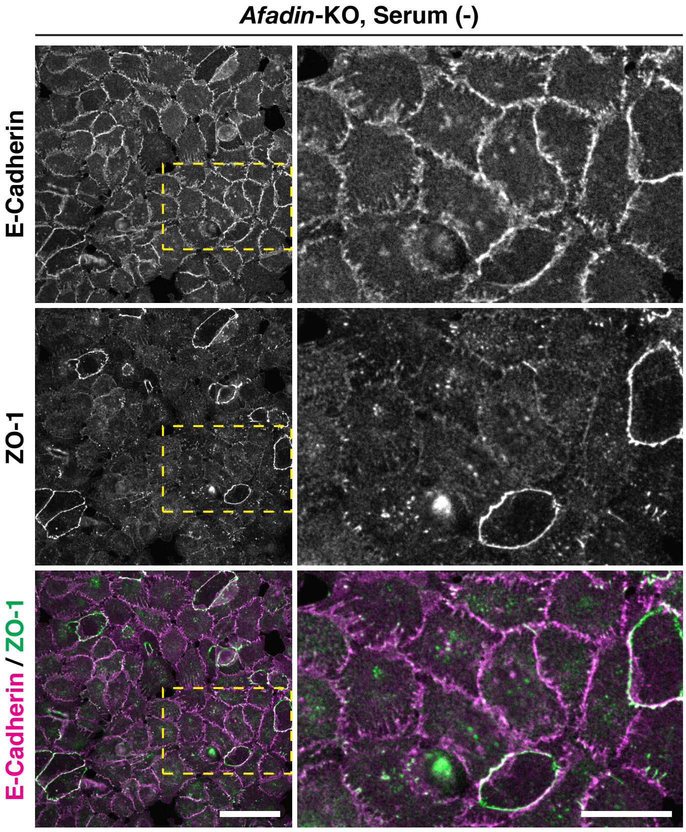

Figure S5. Apparently normal AJC organization in a few *afadin*-KO cells cultured in the serum-free medium. *Afadin*-KO cells were cultured in the serum-free medium for 8 h. The cells were fixed and stained with the indicated Abs, and then observed by immunofluorescence microscopy. The boxed regions in the left panels are highlighted in the right panels. Serum (-) indicates the serum-free medium and scale bars indicate 50 μm for the left panel and 25 μm for the right panel. The results are representative of three independent experiments.

Fig. S6

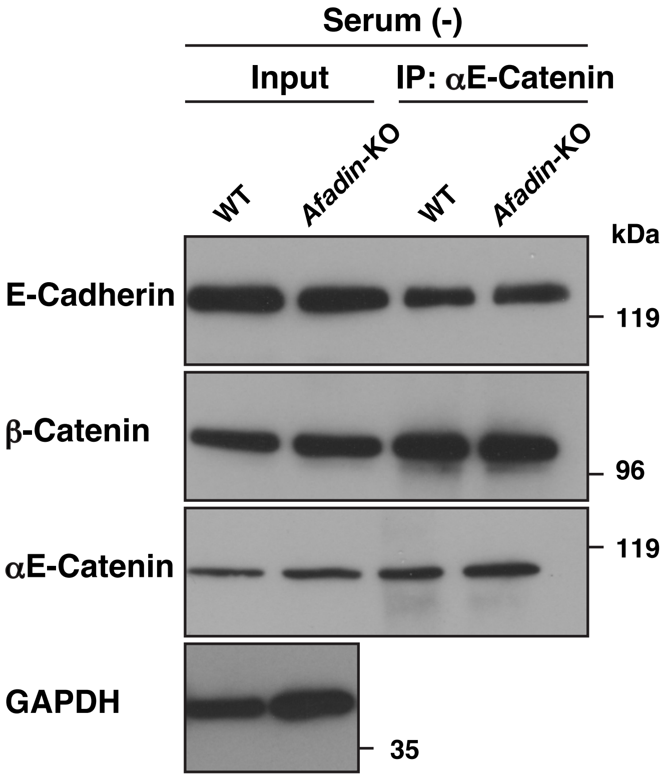

Figure S6. No requirement of afadin or serum for the formation of the αE-catenin–β-catenin–E-cadherin complex at AJs. αE-Catenin was immunoprecipitated with the anti-αE-catenin Ab from the lysates of WT or *afadin*-KO cells cultured in the serum-free medium for 8 h, and the immunoprecipitates were subjected to Western blotting with the indicated Abs. The results are representative of three independent experiments.

Fig. S7

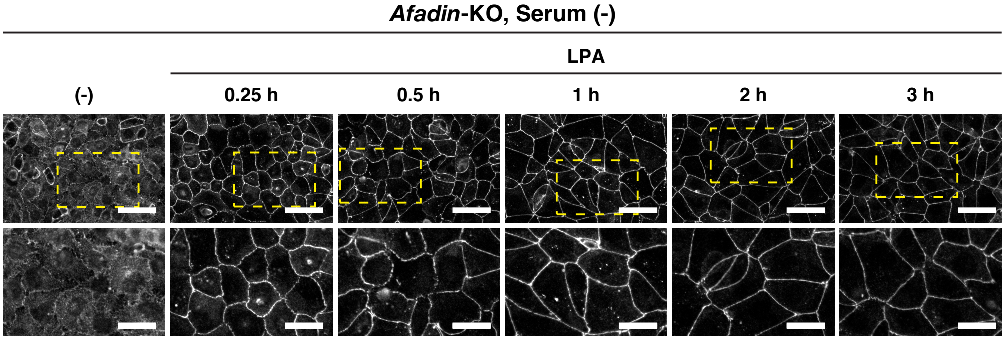

Figure S7. Time-dependent AJC restoration by LPA re-addition in *afadin*-KO EpH4 cells precultured in the serum-free medium. *Afadin*-KO EpH4 cells were precultured in the serum-free medium for 8 h and then cultured in the serum-free medium in the presence of 1% BSA with 10 μM LPA for 0, 0.25, 0.5, 1, 2, or 3 h. The cells were fixed and stained with the anti-ZO-1 Ab, and then observed by immunofluorescence microscopy. The boxed regions in upper panels are highlighted in lower panels. Serum (-) indicates the serum-free medium; and scale bars indicate 50 μm (upper panels) and 25 μm (lower panels). The results are representative of three independent experiments.

Fig. S8

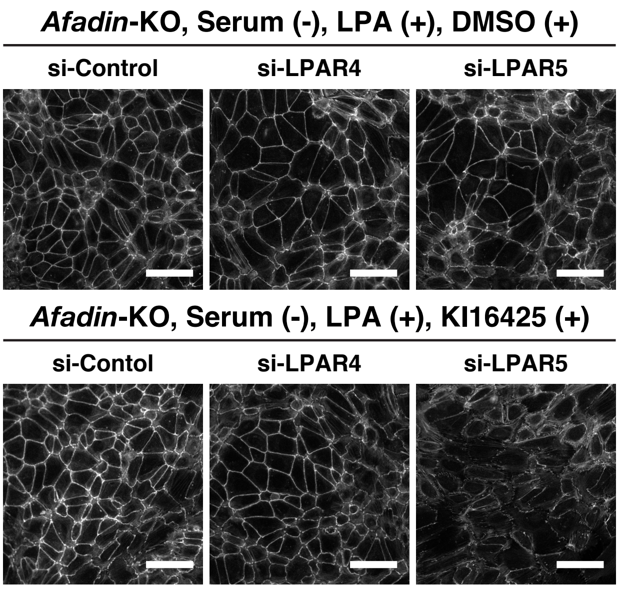

Figure S8. Involvement of LPAR in the LPA-promoted AJC organization. *Afadin*-KO EpH4 cells transfected with control siRNA (si-Control), LPAR4 siRNA (si-LPAR4), or LPAR5 siRNA (si-LPAR5) were cultured in the serum-free medium supplemented with 10 μM LPA in the presence of DMSO or 10 μM KI16425, an LPAR1/3 inhibitor, for 3 h. The cells were fixed and stained with the anti-ZO-1 Ab, and then observed by immunofluorescence microscopy. Scale bars indicate 50 μm. The results are representative of three independent experiments.

Fig. S9

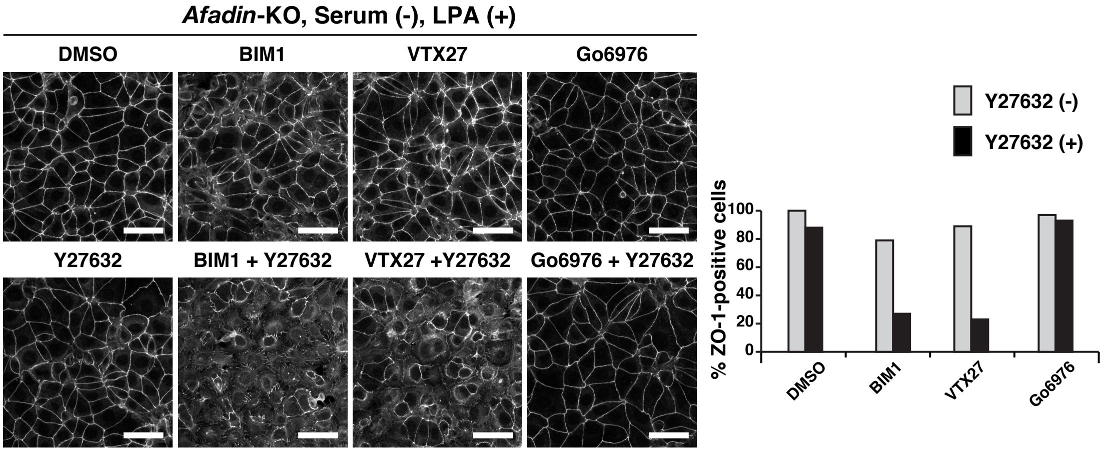

Figure S9. Involvement of nPKC and ROCK activation in the LPA-promoted AJC organization. *Afadin*-KO cells were cultured in the serum-free medium in the presence of 1% BSA and 10 μM LPA together with DMSO or the indicated combinations of inhibitors for 8 h. The cells were fixed and stained with the anti-ZO-1 Ab, and then observed by immunofluorescence microscopy. Quantitative analysis is shown in the right panel. The percentages of the ZO-1-positive cells are 100% (DMSO, n = 191), 79% (BIM1, n = 188), 89% (VTX27, n = 201), 97% (Go6976, n = 198), 88% (Y27632, n = 184), 27% (BIM1/Y27632, n = 193), 23% (VTX27/Y27632, n = 173), and 93% (Go6976/Y27632, n = 207). Serum (-) indicates the serum-free medium; and scale bars indicate 50 μm. The results are representative of three independent experiments.

Table S1. Antibodies used in this study.

Antibody	Use	Dilution	Buffer	Host	Clone #	Catalog #	Supplier	Address
**Primary antibody**								
αE-Catenin	IF, IB	1 / 500, 1/1000	Block Ace, Skim milk	Rb	-	C2081	Sigma-Aldrich	St. Louis, MO, USA
Afadin	IF, IB	1 / 500, 1/1000	Block Ace, Skim milk	Rb	-	A0349	Sigma-Aldrich	St. Louis, MO, USA
β-Catenin	IF	1 / 300	Block Ace	Ms	14/Beta	610154	BD Biosciences	Franklin lakes, NJ, USA
β-Catenin	IF, IB	1 / 500, 1/1000	Block Ace	Rb	-	C2206	Sigma-Aldrich	St. Louis, MO, USA
Claudin-3	IF	1 / 300	Block Ace	Rb	-	34-1700	Thermo Fisher Scientific	Waltham, MA, USA
E-Cadherin	IF	1 / 300	Block Ace	Ms	4A2	14472S	Cell Signaling Technology	Danvers, MA, USA
E-Cadherin	IF, IB	1 / 1000, 1/1000	Block Ace, Skim milk	Rt	ECCD2	Reffered in Methods	-	-
GAPDH	IB	1 / 1000	Skim milk	Ms	6C5	AM4300	Thermo Fisher Scientific	Waltham, MA, USA
Nectin-2	IF	1 / 100	Block Ace	Rt	502-57	D083-3	MBL International	Nagoya, Japan
ZO-1	IF	1 / 300	Block Ace	Ms	ZO1-1A12	33-9100	Thermo Fisher Scientific	Waltham, MA, USA
ZO-1	IF	1 / 300	Block Ace	Rb		61-7300	Thermo Fisher Scientific	Waltham, MA, USA
**Secondary antibody**								
Mouse IgG-Alexa Fluor 488	IF	1 / 500	Block Ace	Gt	-	A11029	Thermo Fisher Scientific	Waltham, MA, USA
Mouse IgG-Alexa Fluor 555	IF	1 / 500	Block Ace	Gt	-	A21424	Thermo Fisher Scientific	Waltham, MA, USA
Mouse IgG-Alexa Fluor 647	IF	1 / 500	Block Ace	Gt	-	A21236	Thermo Fisher Scientific	Waltham, MA, USA
Rat IgG-Alexa Fluor 555	IF	1 / 500	Block Ace	Gt	-	A21434	Thermo Fisher Scientific	Waltham, MA, USA
Rat IgG-Alexa Fluor 647	IF	1 / 500	Block Ace	Gt	-	A21247	Thermo Fisher Scientific	Waltham, MA, USA
Rabbit IgG-Alexa Fluor 488	IF	1 / 500	Block Ace	Gt	-	A11034	Thermo Fisher Scientific	Waltham, MA, USA
Rabbit IgG-Alexa Fluor 555	IF	1 / 500	Block Ace	Gt	-	A21429	Thermo Fisher Scientific	Waltham, MA, USA
Rabbit IgG-Alexa Fluor 647	IF	1 / 500	Block Ace	Gt	-	A21245	Thermo Fisher Scientific	Waltham, MA, USA
Mouse IgG HRP	IB	1 / 10000	Skim milk	Gt	-	115-035-003	Jackson Immunoresearch	West Grove, PA, USA
Rat IgG HRP	IB	1 / 10000	Skim milk	Gt	-	112-035-003	Jackson Immunoresearch	West Grove, PA, USA
Rabbit IgG HRP	IB	1 / 10000	Skim milk	Gt	-	111-035-003	Jackson Immunoresearch	West Grove, PA, USA

IF, immunofluorescence; IB, immunoblotting; Ms, Mouse; Rt, Rat; Rb, Rabbit; Gt, Goat; HRP, horseradish peroxidase.

Table S2. Reagents used in this study.

Reagent	Catalog #	Supplier	Address
**Cell culture and transfection**			
DMEM	08458-16	Nacalai Tesque	Kyoto, Japan
Penicillin-Streptomycin Mixed Solution	26253-84	Nacalai Tesque	Kyoto, Japan
Fetal bovine serum	172012	Sigma-Aldrich	St. Louis, MO, USA
DMEM/F12	11581-15	Nacalai Tesque	Kyoto, Japan
Gentamicin	11980-14	Nacalai Tesque	Kyoto, Japan
Insulin	093-06351	FUJIFILM Wako Pure Chemical Corporation	Osaka, Japan
Trypsin/EDTA Solution	35554-64	Nacalai Tesque	Kyoto, Japan
Lipofectamine 3000	L3000015	Thermo Fisher Scientific	Waltham, MA, USA
Lipofectamine RNAiMAX	13778150	Thermo Fisher Scientific	Waltham, MA, USA
**Activator or inhibitor**			
6-bnz-cAMP	5255	Tocris Bioscience	Bristol, UK
8-br-cGMP	519-40321	Tocris Bioscience	Bristol, UK
bFGF	100-18C	PeproTech	Cranbury, NJ, USA
BIM1	21180	Cayman Chemical Company	Ann Arbor, MI, USA
Blebbistatin	203391	Calbiochem	Billerica, MA, USA
CIAP	2250A	Takara Bio	Kusatsu, Japan
CNF	CN03-A	Cytoskeleton Inc.	Denver, CO, USA
EGF	100-15	Pepro Tech	Cranbury, NJ, USA
IGF-1	100-11	Pepro Tech	Cranbury, NJ, USA
Ionomycin	ab120116	ABCAM LIMITED	Cambridge, UK
Go6976	13310	Cayman Chemical Company	Ann Arbor, MI, USA
KI16425	10012659	Cayman Chemical Company	Ann Arbor, MI, USA
LPA	L7260	Sigma-Aldrich	St. Louis, MO, USA
PA	840875P	Avanti Polar Lipids	Alabaster, AL, USA
S1P	62570	Cayman Chemical Company	Ann Arbor, MI, USA
TGF-β	7754-BH-005	R&D Systems	Minneapolis, MN, USA
TPA	P8139	Sigma-Aldrich	St. Louis, MO, USA
VTX27	HY-112782	MedChem Express	Monmouth Junction, NJ, USA
Y27632	688000	Calbiochem	Billerica, MA, USA
**Cell staining**			
Block Ace	UKB80	KAC	Kyoto, Japan
FluorSave reagent	345789	Merck Millipore	Billerica, MA, USA
Hanks' balanced salt solution	14065056	Thermo Fisher Scientific	Waltham, MA, USA
Normal goat serum	ab7481	ABCAM LIMITED	Cambridge, UK
**RNA purification and Reverse transcription PCR**			
GoTaq DNA Polymerase	M3001	Promega	Madison, WI
RNeasy Mini Kit	74104	Qiagen	Hilden, Germany
SuperScript IV reverse transcriptase	18091050	Thermo Fisher Scientific	Waltham, MA, USA
TRIzol	15596026	Thermo Fisher Scientific	Waltham, MA, USA

Table S3. Sequences of the primers against mouse LPARs, β-actin, and GAPDH.

Gene	Forward	Reverse
Lpar1	5'-CGCCAGAGGACTATGAGGATGT-3'	5'-CAGCAGACAATAAAGGCACCAAG-3'
Lpar2	5'-CCGCTACCGAGAGACCACAC-3'	5'-ACTTACAGTCCAGGCCATCCA-3'
Lpar3	5'-TGCTCATTCTGCTGGTGTGG-3'	5'-TGATGAAGAAGGCCAGGAGGT-3'
Lpar4	5'-ACGGCTATTTTCATCACCAACCT-3'	5'-ATGGCTAGGAAACGATCCACAC-3'
Lpar5	5'-CTGGCTGTATATGGGTTGCTACG-3'	5'-GAAACCCTCGGCACTGAAGTAGT-3'
Lpar6	5'-GGTCATCTTCTGTTTCTGTTTTGTG-3'	5'-TGAGTTCTGAATTGTGTCTGAGGTG-3'
β-Actin	5'-GGCTGTATTCCCCTCCATCGT-3'	5'-AGTTGGTAACAATGCCATGTTCAAT-3'
Gapdh	5'-AGCGAGACCCCACTAACATCAA-3'	5'-TCGTGGTTCACACCCATCAC-3'

Table S4. Sequences of the siRNAs against mouse LPAR4/5 and a negative control siRNA.

**siRNA**	**Target sequence**
Control	5'-CAACAAGATGAAGAGCACCAA-3'
Mouse *Lpar4*	5'-GCAAAGATCATGTACCCAA-3'
Mouse *Lpar5*	5'-CCACTGGTTTACTACTTCA-3'
